# A study to characterize the mechanical properties and material constitution of adult descending thoracic aorta based on uniaxial tensile test and digital image correlation

**DOI:** 10.3389/fbioe.2023.1178199

**Published:** 2023-06-14

**Authors:** Zhengdong Li, Ming Pei, Jianhua Zhang, Ningguo Liu, Jinming Wang, Donghua Zou

**Affiliations:** ^1^ Shanghai Key Laboratory of Forensic Medicine, Key Laboratory of Forensic Science, Ministry of Justice, Shanghai Forensic Service Platform, Academy of Forensic Science, Shanghai, China; ^2^ Institute of Forensic Science, Xuzhou Public Security Bureau, Xuzhou, Jiangsu, China

**Keywords:** human aorta, uniaxial tensile test, digital image correlation, material properties, material constitutive model

## Abstract

The mechanical properties and material constitution of the aorta are important in forensic science and clinical medicine. Existing studies on the material constitution of the aorta do not satisfy the practical requirements of forensic and clinical medicine, as the reported failure stress and failure strain values for human aortic materials have a high dispersion. In this study, descending thoracic aortas were obtained from 50 cadavers (dead within 24 h) free of thoracic aortic disease, aged from 27 to 86 years old, which were divided into six age groups. The descending thoracic aorta was divided into proximal and distal segments. A customized 4-mm cutter was used to punch a circumferential and an axial dog-bone-shaped specimen from each segment; the aortic ostia and calcification were avoided. Instron 8,874 and digital image correlation were used to perform a uniaxial tensile test on each sample. Four samples from each descending thoracic aorta produced ideal stress-strain curves. All parameter-fitting regressions from the selected mathematical model converged, and the best-fit parameters of each sample were obtained. The elastic modulus of collagen fiber, failure stress, and the strain showed a decreasing trend with age, while the elastic modulus of elastic fiber showed an increasing trend with age. The elastic modulus of collagen fiber, failure stress, and strain of circumferential tensile were all greater than those for axial tensile. There was no statistical difference in model parameters and physiological moduli between the proximal and distal segments. The failure stress and strain in the proximal circumferential, distal circumferential, and distal axial tensile were all greater for the male group than for the female group. Finally, the Fung-type hyperelastic constitutive equations were fitted for the different segments in different age groups.

## 1 Introduction

The aorta, the largest blood vessel in the body, is the primary artery in the systemic circulation. It is very important for maintaining the function of the human body. Aorta rupture causes up to 80%–94.4% mortality and is an important cause of in forensic practice (Hiller et al., 2010; Teixeira et al., 2011; Kent, 2014). Aortic ruptures can be caused by trauma, disease, or medical practice. Distinguishing pathologic and traumatic aortic ruptures from one another is one of the difficulties in forensic identifications, especially with regard to the identification of the degree of participation of trauma when multiple trauma exist or trauma and disease coexist. However, the causative mechanism of aorta ruptures, such as deceleration, osseous pinch, torsion, longitudinal stretch, and water-hammer effects, *etc.* (Neschis et al., 2008), are difficult neither to reproduce experimentally on cadavers, nor to observe the dynamic aortic response *in situ* (Bass et al., 2001). The finite element (FE) simulation can be used to study the mechanism of aortic injuries, while mechanical properties and material constitution of the aorta are an important basis of FE simulation, which directly affects the accuracy of simulation results. Clinically, surgical indications of aortic aneurysm mainly depend on the diameter and growth rate (Lederle et al., 2002; Hans et al., 2005; Grootenboer et al., 2009), which ignores the more important relationship between rupture and mechanical properties of the aorta, especially the tensile strength of the aortic wall. In addition, the mechanical properties of the prosthesis for aortic aneurysm surgery should be as consistent as possible with the mechanical properties of the aorta. During interventional surgery, the protection of blood vessels should also take into consideration the material constitution of the walls of the blood vessels. Therefore, the mechanical properties and material constitution of the aorta are very important in forensic and clinical medicine.

At present, the most common methods for studying the mechanical properties of the aorta are uniaxial tensile, biaxial tensile, peeling test, residual strain/stress test. Uniaxial tensile is the main method because it is relatively simple and can be performed on a variety of materials (Raghavan et al., 1996; Sherebrin et al., 1989; Vorp et al., 2003; Vallabhaneni et al., 2004; Guinea et al., 2010; Sokolis et al., 2012; García-Herrera et al., 2012; Ninomiya et al., 2015; Ferrara et al., 2016; Ferrara et al., 2018). We have reviewed the common literature on human aortic stretching experiments and list the results in Table 1. It was found that there are some differences among different studies in the source of inspection materials, geometric shape and size of inspection materials, experimental methods (preloading, pre-stretching, strain rate, clamping, test temperature and environment), strain measurement methods, *etc.* These differences lead to large data discrepancies in different studies, and the material properties of aortas from different parts or even the same part are often inconsistent, and some are even contradictory. In 1982, Mohan and Melvin (Mohan and Melvin, 1982) used a dog-bone-shaped sample with a width of 6.35 mm or 4.57 mm to measure the circumferential and axial failure stress and strain under quasi-static and dynamic conditions. The circumferential and longitudinal tensile failure stresses under quasi-static conditions are 1.72 ± 0.89 MPa and 1.47 ± 0.91 MPa, respectively. Vallabhaneni et al. (Vallabhaneni et al., 2004) reported lower values of 610 ± 70 kPa and 1,300 ± 110 KPa for circumferential and longitudinal failure stresses, respectively, using rectangular samples of healthy thoracic aorta with a width of 4 mm. However, Garcı'a-Herrera et al. (García-Herrera et al., 2012) reported higher values, where the circumferential and longitudinal values can reach 2,180 ± 240 kPa and 1,140 ± 100 kPa. It can be seen from Table 1 that most studies believe that the circumferential stress is greater than the longitudianl stress (Mohan and Melvin, 1982; Raghavan et al., 1996; Sokolis et al., 2012; Pichamuthu et al., 2013), but a small number of studies have the opposite result (Raghavan et al., 2011), especially Vorp et al. (Vorp et al., 2003) considered no difference in material properties in the two directions. The material properties of different segments of the aorta may also be different. Sokolis (Sokolis, 2007) based on the uniaxial tensile test of the porcine aorta, found that under physiological pressure, the material properties and structure of the aorta have segmental changes with different aortic pressures. Haskett et al. (Haskett et al., 2010) found for the first time that the circumferential and axial tangential moduli of the aorta harden with age based on biaxial stretching experiments of the human aorta, and that the abdominal aorta was stiffer than other parts. Furthermore, Sokolis (Sokolis, 2023) studied the failure parameters of nine consecutive segments of the aorta and the intima, media, and adventitia in the circumferential and axial directions. It was found that the strength of the intima and media remained unchanged along the aorta, but its longitudinal stretching ability decreased, and the strength of the adventitia was significantly higher than that of the intima and media. Females rarely have different failure parameters than males. Sokolis has also conducted extensive research on regional delamination strength (Sokolis and Papadodima, 2022), layer-specific residual deformation (Sokolis et al., 2021) in different parts of the aorta, and aortic circumferential residual strains (Sokolis et al., 2017) that vary with sex and age. These studies have helped to advance our understanding of aortic physiology and explain the biomechanism of aortic dissection.

**TABLE 1:** Overview of tensile test results on aorta full-thickness tests performed until failure.

Author	Tissue	Source	Gender	Shape	Dimensions (mm)	Failure rate	Clamping	Stress	Strain	Failure stress (MPa)		Failure strain
Mohan and Melvin (1982)	DTA	AU	-	D	19.05 × 6.35 or 7.87 × 4.57	-	PC	True stress	stretch ratio	Quasi-static	C:1.72 ± 0.89	1.53 ± 0.28
											L:1.47 ± 0.91	1.47 ± 0.91
										Dynamic	C:5.07 ± 3.29	1.60 ± 0.28
											L:3.59 ± 2.04	1.64 ± 0.28
Sherebrin et al. (1989)	DTA	AU	-	R	30 × 5	-	Waterproof sandpaper	True stress	True strain		C:1.77 ± 1.04	0.40 ± 0.16
											L:1.84 ± 0.90	0.31 ± 0.11
Vorp et al. (2003)	ATAA	AU	-	R	30 × 8	-	-	True stress	stretch ratio	ATAA	C:1.18 ± 0.12	-
											L:1.21 ± 0.09	-
	ASA	SU								ASA	C:1.8 ± 0.24	-
											L:1.71 ± 0.14	-
Vallabhaneni et al. (2004)	AAA	AU	-	R	30–40×4	36/184	PC + Silicone Rubber			AAA	L:0.53 ± 0.02	0.3 ± 0.02
	ABA	SU								ABA	C:0.61 ± 0.07	0.29 ± 0.04
											L:1.30 ± 0.11	0.33 ± 0.04
Shah et al. (2006)	A	AU	-	CR	10-mm center square region with 0.9-mm radius between adjacent pairs of branches	-	Tissue clamp	True stress	Lagrange strain	1 -m/s	2.07 ± 1.11	0.26 ± 0.199 (Region of Interest); 0.25 ± 0.125 (Tear Location)
										5 -m/s	1.95 ± 0.89	0.205 ± 0.094 (Region of Interest); 0.26 ± 0.099 (Tear Location)
				-	intact aorta	-	Tissue clamp	Engineering stress	Lagrange strain	Strain rate: 11.8 ± 4.6/s	0.75 ± 0.14	0.221 ± 0.069
Guinea et al. (2010)	DTA	AU	-	D	10 × 2	-	CY	True stress	stretch ratio	16–30 years	C:2.4 ± 0.2	-
											L:1.3 ± 0.1	-
										31–45 years	C:1.3 ± 0.2	-
											L-	-
										46–60 years	C:0.9 ± 0.1	-
											L:0.7 ± 0.1	-
Sokolis et al. (2012)	ATAA	AU	-	R	35 × 10	-	Sandpaper	The second Piola–Kirchhoff stress	stretch ratio/Green strain	ATAA	C:1.663 ± 0.1048	1.52 ± 0.02
											L:1.0675 ± 0.0651	1.52 ± 0.02
	ASA			D	25 × 2					ASA	C:1.6461 ± 0.0484	1.59 ± 0.02
											L:1.0012 ± 0.056	1.64 ± 0.06
García-Herrera et al. (2012)	ASA	AU	-	D	10 × 2	-	CY	True stress	stretch ratio	<35 years	C:2.18 ± 0.24	2.35 ± 0.10
											L:1.14 ± 0.10	2.00 ± 0.10
										>35 years	C:1.20 ± 0.20	-
											L:0.66 ± 0.07	-
	ATAA	SU								BAV	C:1.23 ± 0.15	1.80 ± 0.08
											L:0.84 ± 0.10	1.58 ± 0.06
										ATAA	C:1.19 ± 0.13	-
											L:0.88 ± 0.12	-
Ninomiya et al. (2015)	DTA	AU	Yes	R	40 × 4	64/217	Tissue clamp	True stress	Engineering strain	DTA	C: 1.6879 ± 1.0795	0.66 ± 0.31
	ABA									ABA	C:1.363 ± 0.9537	0.49 ± 0.25
										Posterior	C:1.85 ± 0.70	0.301 ± 0.090
											L:0.75 ± 0.18	0.267 ± 0.065
Sulejmani et al. (2017)	ATAA	SU	-	D	-	-	-	True stress	Green strain	MFS<40 years	C:1.2	0.38
										MFS>40 years	C:0.63	0.08
	TAA/DTA	AU								TAA	C:1.95	0.38
										DTA	C:1	0.13
Ferrara et al. (2018)	ATAA	SU	Yes	D	-	-	PC	True stress	stretch ratio	Anterior	C:1.383 ± 0.630	1.329 ± 0.154
											L:0.832 ± 0.444	1.312 ± 0.150
										Posterior	C:1.681 ± 0.723	1.366 ± 0.162
											L:0.717 ± 0.260	1.296 ± 0.107

Note: A, aorta, ASA, ascending aorta; ATAA, ascending thoracic aortic aneurysms; TAA = thoracic aortic aneurysms; DTA, descending thoracic aorta; ABA, abdominal aorta; AAA = abdominal aortic aneurysms; AU, autopsy; SU, surgery; R, rectangle, D, dog-bone shape; CR, cruciate; CY, cyanopropionate adhesives; PC, pneumatic clamps; C, circumferential stretch; L, longitudinal stretch; BAV, bicuspid aortic valve.

However, the specimen extraction methods and experimental methods used in the above-mentioned studies are not uniform, which has resulted in large differences in the experimental results and a lack of direct comparability. To address this issue, our research group has developed a series of sampling molds and experimental methods suitable for aortic uniaxial tension experiments using porcine aorta. Specifically, the specimens are expanded into a dog-bone shape with a region of interest of 24 mm × 4 mm. Strain is measured using digital image correlation (DIC) (Pei et al., 2021). Due to the small area of the ascending aorta and aortic arch, we were unable to extract enough specimens from these regions, which could have affected the systematicness of the data. Additionally, among patients with blunt chest trauma, the most common site of aortic rupture is the isthmus, followed by the distal descending aorta, ascending aorta, and aortic arch (Watanabe et al., 2013). For these reasons, we performed material sampling and tensile testing on the part below the aortic isthmus.

The reported failure stress and failure strain values for human aortic materials have a high dispersion. This, along with the lack of systematic and large-sample Chinese experimental data, means that the current state of knowledge is not sufficient to satisfy the needs of forensic science and clinical medicine in China. Therefore, it is necessary to conduct systematic material property testing on Chinese human aorta. In this study, 50 adult descending thoracic aortas were divided into six age groups, and the descending thoracic aorta was divided into proximal and distal segments. A customized cutter was used to punch a circumferential and an axial dog-bone-shaped specimen in each segment, while avoiding the aortic ostia and calcification. Instron 8,874 and DIC were used to perform a uniaxial tensile test on each sample. The mechanical properties and hyperelastic material constituents of different parts of the descending thoracic aorta from each age group were characterized. Finally, the differences between age groups, sex, tensile direction, and segments were compared and analyzed.

## 2 Materials and methods

### 2.1 Specimen collection and initial processing

This study was approved by the Ethics Committee of the Academy of Forensic Science (Ministry of Justice, China), including the acquisition of specimens and informed consent of close relatives of the deceased. Fresh corpses in 50 forensic cases (dead within 24 h and without thoracic aortic aneurysm, aortic dissection and other serious diseases) were recruited ranging in age from 27 to 86 years. The average age was 51.14 years, and the median age was 51.5 years old. There were 34 men and 16 women, divided into six age groups (The group 1 consisted of 13 corpses, aged 27–35 years old. Of these, 11 were male and 2 were female. The group 2 consisted of 9 corpses, aged 36–45 years old. Of these, 7 were male and 2 were female. The group 3 consisted of 7 corpses, aged 46–55 years old. Of these, 3 were male and 4 were female. The group 4 consisted of 11 corpses, aged 56–65 years old. Of these, 6 were male and 5 were female. The group 5 consisted of 5 corpses, aged 66–75 years old. Of these, 3 were male and 2 were female. The group 6 consisted of 5 corpses, aged 76–86 years old. Of these, 4 were male and 1 was female.). The descending thoracic aorta was extracted from cadavers.

After dissections, the samples were stored at −80°C, and the tests were completed within 1 week. Before the experiment, the sample was taken from the −80°C refrigerator and soaked overnight in a normal saline solution without Ca^2+^. After equilibrium at room temperature, the loose connective tissue attached to the adventitia of the aorta was dissected. The human descending thoracic aorta was divided into proximal and distal segments at the level of the seventh-eighth intercostal posterior artery. Circumferential and axial are defined according to the direction of aortic curvature. A customized 4-mm cutter (Pei et al., 2021) (see in Supplementary Figure S1) was used to systematically punch out two circumferential (upper part) and two axial (lower part) dog-bone-shaped samples from each segment (see in Supplementary Figure S2). The dimensions of the narrow middle part were 24 mm × 4 mm [optimal dimensions identified in our previous study (Pei et al., 2021)]; moreover, the calcification and aortic ostia were avoided.

The original thickness and width of the test area were photographed under the unloaded state with a customized profile measurement system (see in Supplementary Figure S3). The VisionMaster3 software (Hikrobot Co., Ltd., China) was used for measurements. After the profile was measured, black speckles were sprayed on the endothelial surface of the test area. The convex plates of the customized clamps were placed in the fixture groove, and the middle part of the specimen was placed in the specimen groove of the customized mold. Subsequently, the two ends of the specimen were quickly squeezed between the clamps and using cyanoacrylate to avoid slippage (Pei et al., 2021) (the photo of sample in the grips of customized apparatus is shown in Supplementary Figure S4).

### 2.2 Uniaxial tension

The samples were subjected to uniaxial tensile tests using an Instron 8,874 (Illinois Tool Works Inc., USA) coupled to a DIC system at room temperature. The clamps and samples were mounted in the jaws of the pneumatic grips of the Instron 8,874. The distance between the grips was adjusted, starting from a slightly curved configuration. The sample was then slowly extended until the load cell recorded the minimum tensile force, which was assumed to be the load-free configuration (initial point). The distance between the grips at this initial point was denoted as the original length L). Each specimen was preconditioned at a speed of 0.2 L per minute and a stretching amplitude of 0.04 L for five cycles to eliminate the hysteresis effect and obtain a repeatable stress-strain curve. The specimen was then stretched at the same speed until it failed. The applied force was continuously measured and stored by Instron 8,874 (maximum load of 25 kN with an accuracy of 0.5% of full scale), and the DIC system (with resulotion of 5 million pixels) synchronously collected surface strain photos of the specimens at a sampling rate of 1 Hz to record the strain in the tensile direction. Physiological saline was sprayed on the specimens to keep them moist before and during the experiment.

## 3 Data analysis

### 3.1 Selection and calculation of the mechanical parameters

According to the width and thickness measured for the initial state of the specimens, combined with the incompressible nature of the aorta (Chuong and Fung, 1983) (the volume of the specimen is constant during the stretching process), engineering strain and stress, stretch ratio, true strain, and true stress can be calculated and converted.

The engineering strain ( $\varepsilon_{E}$ ) is equal to the change in length (*ΔL*) divided by the initial length (*L*_*0*_) of the specimen test area:

$$\varepsilon_{E}=\frac{\Delta L}{L_{0}}$$

The true stress ( $\sigma_{T}$ ) is expressed by the applied load *F*) divided by the current cross-sectional area *A*), *A*_*0*_ is the original cross-sectional area:

$$\sigma_{T}=\frac{F}{A}=\frac{F\times \left(\Delta L+L_{0}\right)}{A_{0}\times L_{0}}=\frac{F}{A_{0}}\left(1+\varepsilon_{E}\right)$$

In order to simplify the analysis and facilitate comparison between different groups of specimens, this study adopted the mathematical model proposed by Raghavan et al. (Raghavan et al., 1996):

$$\varepsilon =\left(K+\frac{A}{B+\sigma }\right)\sigma$$

In this model, ε is the engineering strain; σ is the true stress; *K*, *A,* and *B* are model parameters. According to this theory, the aortic wall is assumed to contain only two main passive load-bearing fibers: elastic and collagenous fibers. The stress-strain curve can be divided into three phases [As shown in study of Pei et al. (Pei et al., 2021)].

In the first phase, the tensile stress is low and only elastic fibers bear the tensile stress. At this time, σ → 0, *B*+ σ ≈ *B*, the $\varepsilon =\left(K+\frac{A}{B+\sigma }\right)\sigma$ becomes $\varepsilon =\left(K+\frac{A}{B}\right)\sigma$ , and the elastic modulus of the elastic fiber (*E*_*E*_) can be expressed as:

$$E_{E}=\frac{\sigma }{\varepsilon }=\frac{1}{K+A/B}$$

In phase 2, collagen fibers begin to bear the tensile load, and the slope of the stress-strain curve increases, indicating hyperelasticity.

In phase 3, all the collagen fibers have become taut and the stiffness of the tissue reaches its maximum. Here, the slope of the stress-strain curve corresponds to the sum of the total stiffness of the elastic and collagen fibers. As *B* ≪σ , *B*+ σ ≈ σ , the formula of $\varepsilon =\left(K+\frac{A}{B+\sigma }\right)\sigma$ becomes $\varepsilon =\left(K+\frac{A}{\sigma }\right)\sigma$ . The sum of the elastic modulus of the elastic and collagen fibers and the elastic modulus of collagen fiber (*E*_*C*_) can be deduced as:

$$E_{E}+E_{C}=\frac{1}{K}$$

$$E_{C}=\frac{A}{K\left(A+KB\right)}$$

According to the above formula, *K* is the inverse of (*E*_*E*_ + *E*_*C*_). *A* is the strain intercept of the maximum slope (*E*_*E*_ + *E*_*C*_) of the stress-strain curve during the third phase. A is related to the recruitment speed of collagen fibers. The smaller the *A* value, the faster the collagen fibers bear the load. *B* is the stress value at the intersection of the slope of phase 1 and the maximum slope of phase 3. *σ*_*u*_ and *ε*_*u*_ are failure stress (the true stress) and failure strain (the engineering strain), respectively. In summary, the stress-strain curve is simplified as a function of three parameters: *K*, *A,* and *B*. *E*_*E*_ and *E*_*C*_ can be derived from those parameters.

On the basis of the above analysis, the Fung-type strain-energy function (Chuong and Fung, 1983) was used to fit the average stress-strain curve for the original data from each age group to characterize the mechanical properties of the descending thoracic aorta. This strain-energy function is the most commonly used two-dimensional exponential model in biomechanics literature regarding soft tissue (Sacks and Sun, 2003; Vito and Dixon, 2003; Sokolis et al., 2012). In this model, the aorta is regarded as incompressible, homogeneous, nonlinear, and anisotropic, and the function is expressed as:

$$W=K\left(e^{Q}-1\right),Q=C_{\theta \theta }E_{\theta }^{2}+C_{ZZ}E_{Z}^{2}+C_{\theta Z}E_{\theta }E_{Z}$$

where *E*_*i*_ is Green strain, which is calculated as:

$$E_{i}=\frac{1}{2}\left(\lambda_{i}^{2}-1\right)$$

*λ* is the stretch ratio; *i* = *θ*, *z* which represent the circumferential and axial tensile directions, respectively. The material constant *K* is a scale factor, and *C*_*θθ*_ *, C*_*zz*_, and *C*_*θz*_ represent the circumferential stiffness, axial stiffness, and the interaction between circumferential and axial stiffness, respectively.

The formula to derive Cauchy stress (the true stress) is:

$$\sigma_{i}=\lambda_{i}^{2}\frac{\partial W}{{\partial E}_{i}}$$

The boundary condition of circumferential stretching is: $\sigma_{Z}=0\Rightarrow 2C_{ZZ}E_{Z}+C_{\theta Z}E_{\theta }=0$

The boundary condition of axial stretching is: $\sigma_{\theta }=0\Rightarrow 2C_{\theta \theta }E_{\theta }+C_{\theta Z}E_{Z}=0$

### 3.2 Data processing and statistical analyses

The SPSS software (version 20) was used for parameter fitting and statistical analyses. According to the data collected and calculated by the material testing and DIC systems, as well as the original cross-sectional area of specimens, the stress-strain curve, failure stress, and failure strain were obtained for each specimen. The nonlinear regression Levenberg–Marquardt algorithm was used to fit Raghavan’s mathematical model with the stress-strain data from each sample, and the best-fitting parameters *K*, *A,* and *B* were obtained for each sample. The elastic modulus of the elastic fiber *E*_*E*_ and the elastic modulus of the collagen fiber *E*_*C*_ were then calculated. The Levenberg–Marquardt minimization algorithm was also used to fit the average stress-strain curves for the original data from each age group, and the Fung-type strain-energy function was fitted. The average stress-strain curves for circumferentially and axially oriented tissue were fitted concurrently using the Fung-type model.

The root mean square difference *ε*) and correlation coefficient (*R* ^*2*^) were used to evaluate the fitting degree of the experimental data and the model. Calculation methods for this are presented below:

$$\varepsilon =\sqrt{\frac{\sum_{µ=1}^{m}{\left(\sigma_{\theta µ}^{\exp}-\sigma_{\theta µ}^{\mathrm{mod}}\right)}^{2}+\sum_{v=1}^{n}{\left(\sigma_{zv}^{\exp}-\sigma_{zv}^{\mathrm{mod}}\right)}^{2}}{m+n-4}}$$

$$R^{2}=1-\frac{\sum_{µ=1}^{m}{\left(\sigma_{\theta µ}^{\exp}-\sigma_{\theta µ}^{\mathrm{mod}}\right)}^{2}+\sum_{v=1}^{n}{\left(\sigma_{zv}^{\exp}-\sigma_{zv}^{\mathrm{mod}}\right)}^{2}}{\sum_{µ=1}^{m}{\left(\sigma_{\theta µ}^{\exp}\right)}^{2}+\sum_{v=1}^{n}{\left(\sigma_{zv}^{\exp}\right)}^{2}-{\left(\sum_{µ=1}^{m}\sigma_{\theta µ}^{\exp}+\sum_{v=1}^{n}\sigma_{zv}^{\exp}\right)}^{2}/\left(m+n\right)}$$

Where exp and mod represent experimental and model data; *θ* and *z* represent circumferential and axial tensile directions, respectively, and *m* and *n* are the number of data points tested in the two directions respectively (Iliopoulos et al., 2013). *ε* ≤ 0.1 means good fitting to the experimental data.

According to the previous grouping, the values of each parameter are expressed as the mean ± standard deviation of each group. ANOVA was used for comparison between the groups, and LSD (least significant difference) was used for multiple comparison tests if the overall comparison was different. Comparison between the different segments (in all individuals), genders (in the third, fourth, and fifth groups, with a relatively appropriate sex ratio and quantity) and two directions was performed by a two-tailed independent *t*-test. A significant difference was assumed if a *p*-value was less than 0.05.

## 4 Results

### 4.1 The tested stress-strain curves of human aortas

**FIGURE 1:**
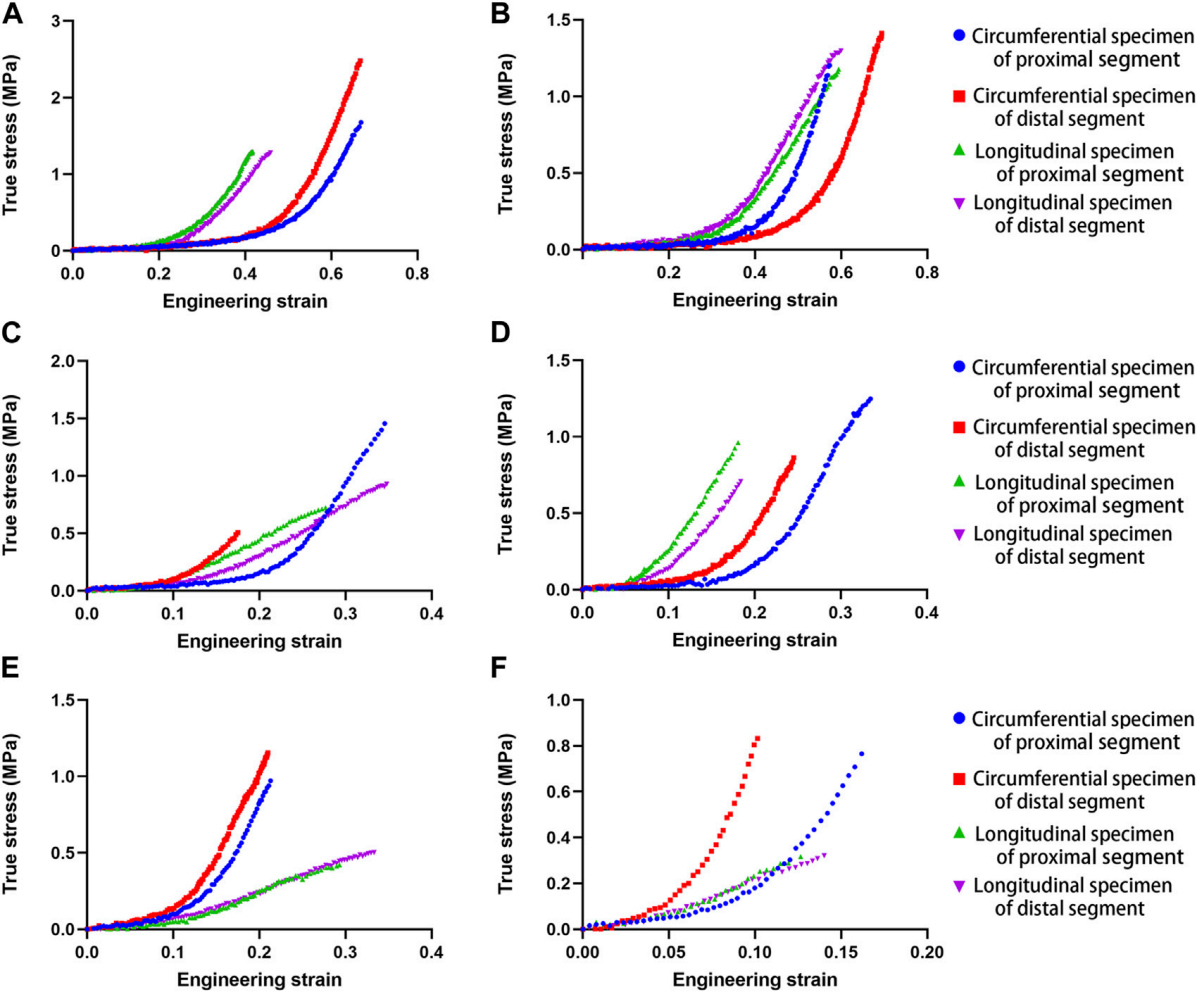
Uniaxial tensile stress-strain curves in human aortas at different ages **(A)**. 28 years; **(B)**. 37 years; **(C)**. 54 years; **(D)**. 64 years; **(E)**. 75 years; **(F)**. 81 years.

Ideal stress-strain curves were obtained from four specimens of each descending thoracic aorta. Figure 1 shows the stress-strain curves of four samples from each representative aorta from the different age groups, including proximal and distal circumferential and axial stretching. Rapidly increasing elastic modulus in the stress-strain curves indicates a significant hyperelastic characteristic of the human aorta, and the increase in the elastic modulus of circumferential specimens is more obvious than that of the axial specimens. The failure stress and strain of circumferential specimens are larger than that of axial specimens. With increasing age, the failure stress and strain tend to decrease.

### 4.2 Parameter fitting and physiological moduli

**FIGURE 2:**
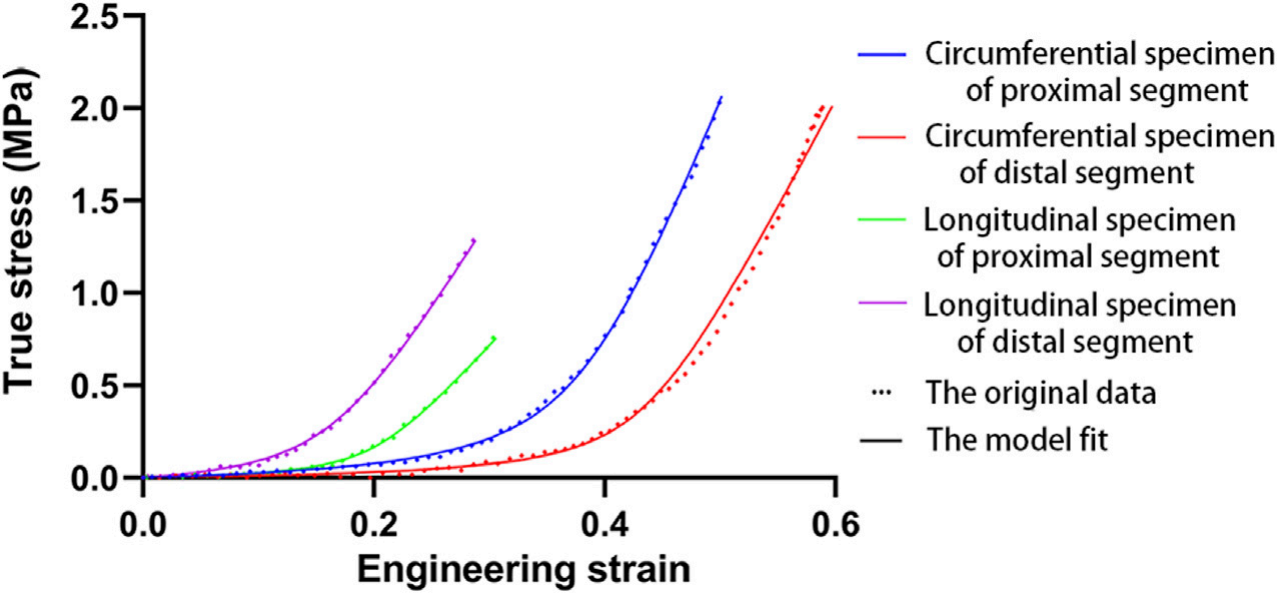
The original data and model fitting (35 years).

**FIGURE 3:**
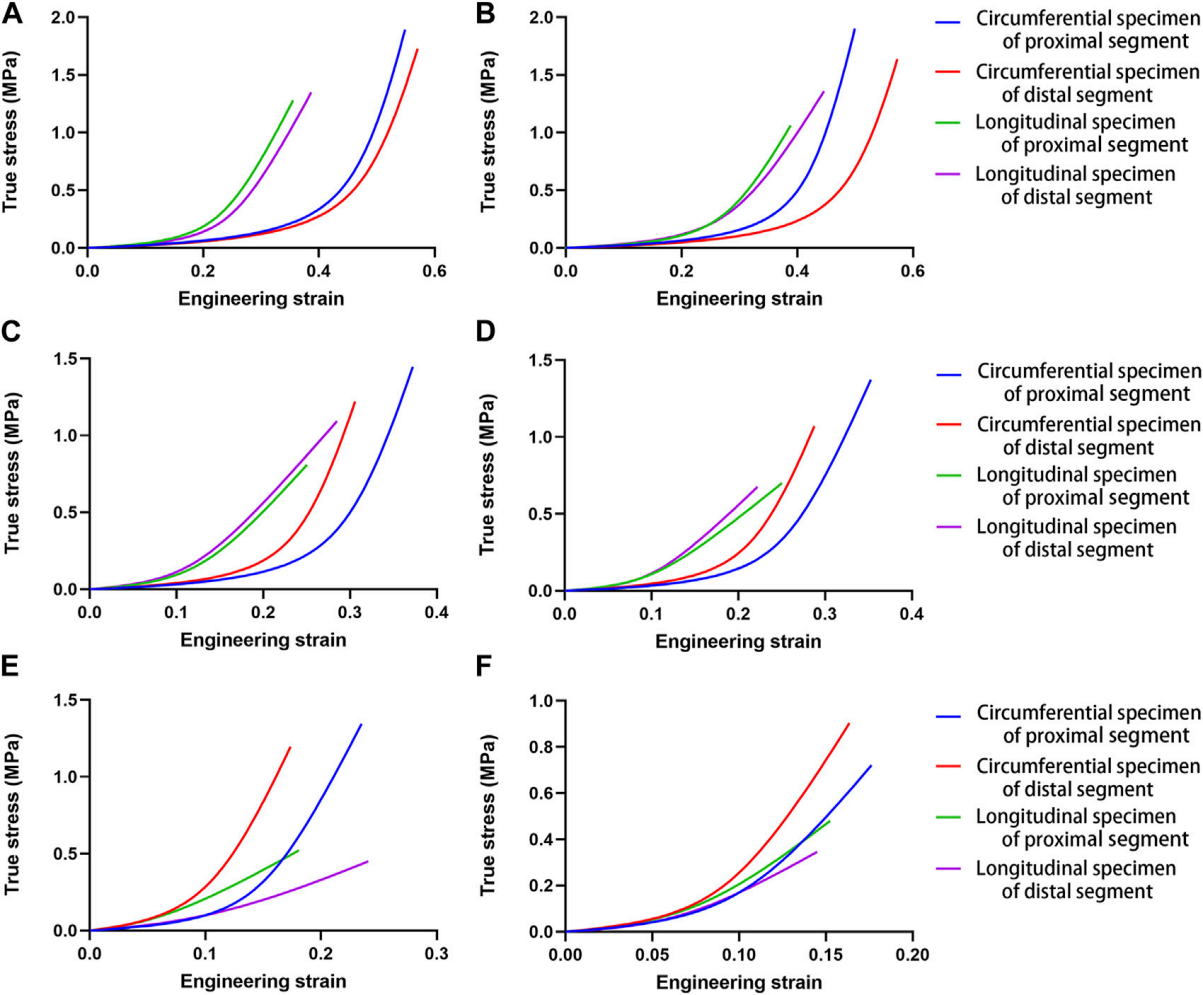
Mean stress-strain curves for the different tensile directions in different aortic segments for the different age groups. **(A)**. Group 1, 27–35 years; **(B)**. Group 2, 36–45 years; **(C)**. Group 3, 46–55 years; **(D)**. Group 4, 56–65 years; **(E)**. Group 5, 66–75 years; **(F)**. Group 6, 76–86 years.

All parameter-fitting regressions of the selected mathematical model in this study converge to obtain the best-fitting parameters for each sample. The stress-strain curves of all specimens indicate a satisfactory fit with the mathematical model. Figure 2 shows the original experimental data for the four specimens of one aorta and the mathematical model curves with the best-fitting parameters. The mean values of parameters for the different parts and different age groups are shown in Table 2, and the mean values of the physiological moduli are shown in Table 3. The average stress-strain curves for the different tensile directions of the different parts and different age groups are plotted in Figure 3 using the average values of model parameters (Table 2).

**TABLE 2:** The mean model parameters for the different aortic parts for the different age groups.

			Circumferential tension			Axial tension		
Group	N	Segment	*K* (mm^2^/N)	*A*	*B* (MPa)	*K* (mm^2^/N)	*A*	*B* (MPa)
27–35 years	13	Proximal segment	0.040 ± 0.021	0.497 ± 0.166	0.096 ± 0.025	0.094 ± 0.022	0.247 ± 0.074	0.066 ± 0.024
		Distal segment	0.047 ± 0.015	0.515 ± 0.142	0.091 ± 0.031	0.101 ± 0.027	0.261 ± 0.069	0.057 ± 0.023
36–45 years	9	Proximal segment	0.040 ± 0.026	0.441 ± 0.085	0.079 ± 0.030	0.104 ± 0.042	0.294 ± 0.149	0.061 ± 0.039
		Distal segment	0.045 ± 0.034	0.524 ± 0.063	0.081 ± 0.036	0.113 ± 0.046	0.309 ± 0.092	0.076 ± 0.027
46–55 years	7	Proximal segment	0.051 ± 0.027	0.313 ± 0.043	0.070 ± 0.023	0.150 ± 0.072	0.138 ± 0.064	0.057 ± 0.030
		Distal segment	0.050 ± 0.025	0.258 ± 0.075	0.066 ± 0.030	0.149 ± 0.071	0.129 ± 0.042	0.062 ± 0.033
56–65 years	11	Proximal segment	0.071 ± 0.047	0.266 ± 0.103	0.058 ± 0.032	0.210 ± 0.138	0.110 ± 0.057	0.046 ± 0.043
		Distal segment	0.064 ± 0.030	0.232 ± 0.053	0.063 ± 0.022	0.178 ± 0.070	0.108 ± 0.050	0.040 ± 0.019
66–75 years	5	Proximal segment	0.063 ± 0.013	0.158 ± 0.070	0.069 ± 0.017	0.222 ± 0.142	0.075 ± 0.041	0.081 ± 0.101
		Distal segment	0.055 ± 0.012	0.118 ± 0.041	0.114 ± 0.031	0.288 ± 0.211	0.132 ± 0.070	0.085 ± 0.041
76–86 years	5	Proximal segment	0.102 ± 0.047	0.111 ± 0.048	0.058 ± 0.042	0.157 ± 0.104	0.086 ± 0.041	0.056 ± 0.053
		Distal segment	0.076 ± 0.035	0.102 ± 0.036	0.068 ± 0.025	0.220 ± 0.067	0.076 ± 0.062	0.036 ± 0.023
All	50	Proximal segment	0.057 ± 0.037	0.333 ± 0.167	0.074 ± 0.031	0.148 ± 0.100	0.177 ± 0.116	0.060 ± 0.045
		Distal segment	0.055 ± 0.027	0.337 ± 0.189	0.080 ± 0.032	0.157 ± 0.098	0.186 ± 0.108	0.058 ± 0.030

Note: Values are presented as mean ± standard deviation.

**TABLE 3:** Mean physiological modulus of the different parts for the different age groups.

			Circumferential tension				Axial tension			
Group	N	Segment	*E*_*E*_ (MPa)	*E*_*C*_ (MPa)	*ε*_*u*_	*σ*_*u*_ (MPa)	*E*_*E*_ (MPa)	*E*_*C*_ (MPa)	*ε*_*u*_	*σ*_*u*_ (MPa)
27–35 years	13	Proximal segment	0.218 ± 0.086	31.948 ± 15.903	0.534 ± 0.153	1.892 ± 0.551	0.271 ± 0.094	10.892 ± 2.618	0.352 ± 0.075	1.276 ± 0.477
		Distal segment	0.179 ± 0.056	23.050 ± 8.188	0.569 ± 0.134	1.711 ± 0.544	0.215 ± 0.064	10.327 ± 2.719	0.385 ± 0.078	1.343 ± 0.517
36–45 years	9	Proximal segment	0.175 ± 0.053	37.920 ± 25.642	0.498 ± 0.054	1.727 ± 0.374	0.493 ± 0.922	11.612 ± 7.855	0.394 ± 0.172	1.076 ± 0.186
		Distal segment	0.157 ± 0.081	32.587 ± 18.065	0.582 ± 0.091	1.621 ± 0.395	0.267 ± 0.138	10.181 ± 4.883	0.440 ± 0.111	1.371 ± 0.436
46–55 years	7	Proximal segment	0.225 ± 0.084	23.963 ± 10.432	0.367 ± 0.050	1.430 ± 0.606	0.404 ± 0.157	7.512 ± 2.997	0.243 ± 0.056	0.803 ± 0.210
		Distal segment	0.296 ± 0.220	26.416 ± 18.441	0.311 ± 0.113	1.221 ± 0.537	0.458 ± 0.197	7.432 ± 2.939	0.239 ± 0.068	0.882 ± 0.332
56–65 years	11	Proximal segment	0.242 ± 0.209	17.363 ± 6.953	0.334 ± 0.104	1.358 ± 0.699	0.355 ± 0.173	5.551 ± 2.158	0.227 ± 0.067	0.682 ± 0.266
		Distal segment	0.275 ± 0.097	18.180 ± 6.969	0.287 ± 0.051	1.061 ± 0.230	0.440 ± 0.361	5.999 ± 2.371	0.216 ± 0.063	0.675 ± 0.198
66–75 years	5	Proximal segment	0.473 ± 0.192	15.733 ± 2.707	0.234 ± 0.043	1.358 ± 0.524	0.898 ± 0.889	6.045 ± 4.592	0.181 ± 0.068	0.607 ± 0.329
		Distal segment	1.050 ± 0.587	17.753 ± 3.195	0.171 ± 0.039	1.184 ± 0.360	0.653 ± 0.501	4.690 ± 3.039	0.233 ± 0.107	0.572 ± 0.131
76–86 years	5	Proximal segment	0.509 ± 0.218	14.553 ± 14.758	0.174 ± 0.054	0.739 ± 0.240	0.567 ± 0.322	7.408 ± 3.120	0.149 ± 0.040	0.507 ± 0.174
		Distal segment	0.742 ± 0.402	14.440 ± 5.749	0.162 ± 0.062	0.912 ± 0.303	0.555 ± 0.315	4.299 ± 1.454	0.138 ± 0.043	0.348 ± 0.106
All	50	Proximal segment	0.271 ± 0.178	25.335 ± 16.952	0.394 ± 0.157	1.511 ± 0.621	0.440 ± 0.506	8.540 ± 4.737	0.279 ± 0.124	0.899 ± 0.415
		Distal segment	0.356 ± 0.365	22.776 ± 12.601	0.393 ± 0.193	1.350 ± 0.504	0.386 ± 0.293	7.777 ± 3.815	0.297 ± 0.129	0.960 ± 0.513

Note: Values are presented as mean ± standard deviation.

### 4.3 Comparison of fitting parameters between the different age groups

**FIGURE 4:**
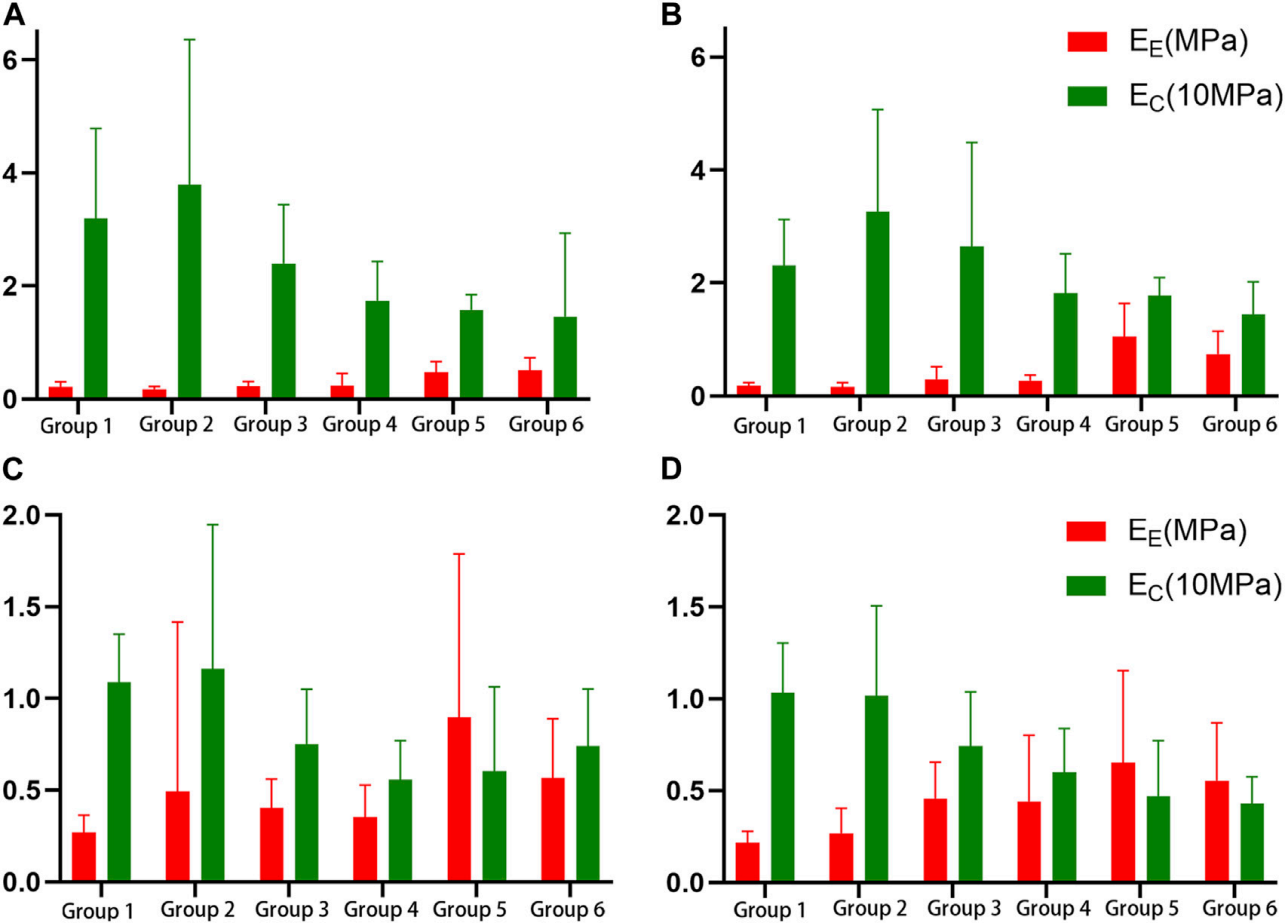
Histogram of the elastic fiber modulus (*E*_*E*_) and the collagen fiber modulus (*E*_*C*_). **(A)**. Proximal circumferential tensile; **(B)**. Distal circumferential tensile; **(C)**. Proximal axial tensile; **(D)**. Distal axial tensile.

**FIGURE 5:**
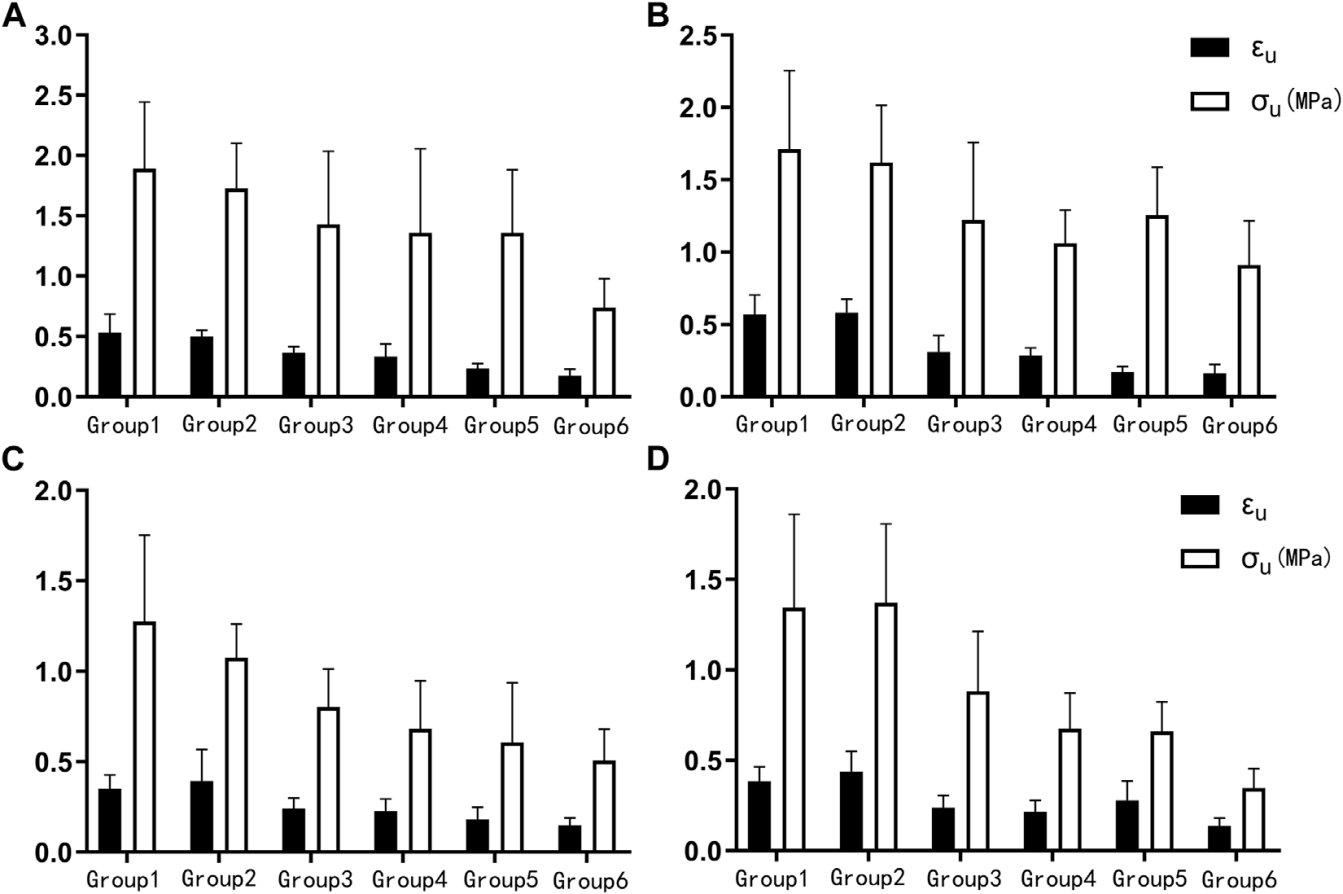
Histogram of the failure stress (*σ*_*u*_) and failure strain (*ε*_*u*_). **(A)**. Proximal circumferential tensile; **(B)**. Distal circumferential tensile; **(C)**. Proximal axial tensile; **(D)**. Distal axial tensile.

**FIGURE 6:**
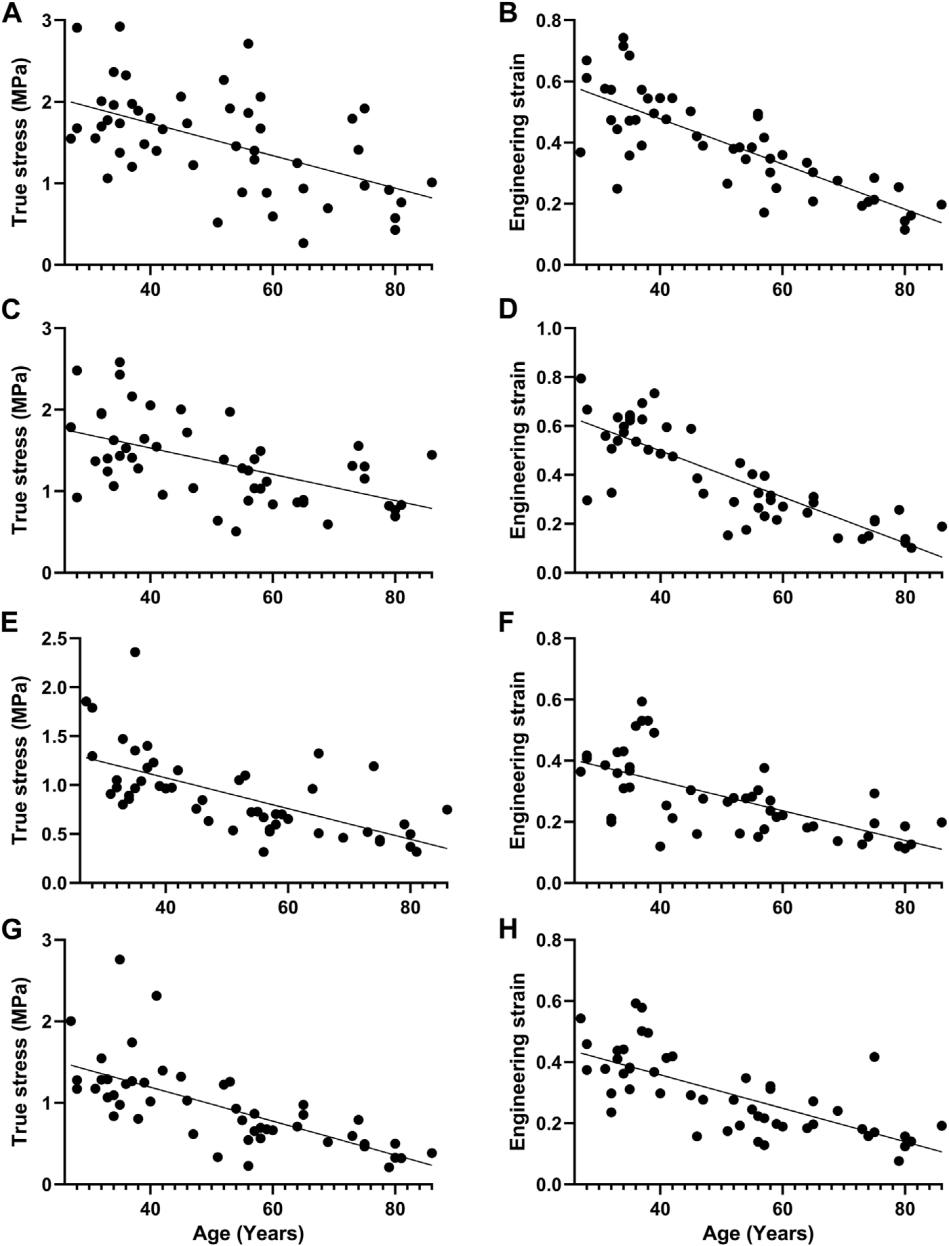
Scatter plot of failure stress and failure strain with age and straight line fitting. **(A, B)**. Proximal circumferential tensile; **(C,D)**. Distal circumferential tensile; **(E,F)**. Proximal axial tensile; **(G,H)**. Distal axial tensile.

*K*, *A*, *B*, *E*_*E*_, *E*_*C*_, the failure stress and the failure strain were different among the proximal circumferential tensile groups. The pairwise comparison results are listed below. *K*: there was a statistical difference between groups 1, 2 and 4, 6, and a statistical difference between groups 3 and 6. *A*: there was a statistical difference between groups 1, 2 and 3, 4, 5, 6. *B*: there was a statistical difference between groups 1 and 4, 6. *E*_*E*_ increased with age, and there was a statistical difference between groups 1, 2, 3, 4, and 5, 6. *E*_*C*_ decreased with age, and there was a statistical difference between groups 1, 2 and 4, 6 (Figure 4A). The failure stress decreased with age, and there was a statistical difference between groups 1 and 4, 6, and a statistical difference between groups 2, 3, 4, and 6. The failure strain decreased with age, and there were statistical differences between groups 1, 2 and 3, 4, 5, 6, and between 3 and 5, 6, and between 4 and 6 (Figure 5A). Figures 6A,B show the scatter plots of failure stress and the failure strain with age. The linear fitting equations were *σ*_*u*_ = 2.536–0.02 × Age, *R* ^2^ = 0.296, and *ε*_*u*_ = 0.774–0.007 × age, *R* ^2^ = 0.629.

*A*, *B*, *E*_*E*_, failure stress, and failure strain were different among the groups under distal circumferential tensile. The pairwise comparison results are listed below. *A*: there were statistical differences between groups 1, 2 and 3, 4, 5, 6, and between 3, 4 and 5, 6. *B*: there were statistical differences between groups 1 and 4 and between 2, 3, 4, 6 and 5. *E*_*E*_ increased with age, and there were statistical differences between groups 1, 2, 3, 4 and 5, 6, and between 5 and 6 (Figure 4B). The failure stress decreased with age, and there were statistical differences between groups 1 and 3, 4, 5, 6, and between 2, and 4, 6. The failure strain decreased with age, and there were statistical differences between groups 1, 2 and 3, 4, 5, 6, and between 3, 4 and 5, 6 (Figure 5B). Figures 6C,D show the scatter plots of failure stress and the failure strain with age. The linear fitting equation is *σ*_*u*_ = 2.174–0.016 × age, *R* ^2^ = 0.294, and *ε*_*u*_ = 0.876–0.009 × age, *R* ^2^ = 0.691.

*K*, *A*, *E*_*C*_, failure stress, and failure strain were different among the groups under proximal axial tensile. The pairwise comparison results are listed below. *K*: groups 1, 2 were statistically different from 4, 5. *A*: groups 1, 2 were statistically different from 3, 4, 5, 6. *E*_*C*_ decreased with age, and there was a statistical difference between groups 1, 2 and 4, 5 (Figure 4C). The failure stress decreased with age, and there were statistical differences between groups 1 and 3, 4, 5, 6, and between 2 and 4, 5, 6. The failure strain generally showed a decreasing trend with age, and there was a statistical difference between groups 1, 2 and 3, 4, 5, 6 (Figure 5C). Figures 6E,F show the scatter plots of failure stress and failure strain with age. The linear fitting equation is *σ*_u_ = 1.704–0.016 × age, *R* ^2^ = 0.413, and *ε*_*u*_ = 0.528–0.005 × age, *R* ^2^ = 0.441.

*K*, *A*, *B*, *E*_*E*_, *E*_*C*_, failure stress, and failure strain were different among the groups under distal axial tensile. Pairwise comparison, *K*: there were statistical differences between groups 1 and 4, 5, 6, between 2 and 5, 6, and between 3, 4 and 5. *A*: groups 1, 2 were statistically different from 3, 4, 5, 6. *B*: there were statistically significant differences between groups 1 and 5, between 2 and 4, 6, between 3 and 6, between 4 and 5, and between 5 and 6. *E*_*E*_ increased with age, and there was a statistical difference between groups 1, 2 and 4, 5, 6. *E*_*C*_ decreased with age, and there was a statistical difference between groups 1, 2 and 4, 5, 6 (Figure 4D). The failure stress showed a decreasing trend with age, and groups 1, 2 were statistically different from the other four groups, and group 3 was statistically different from 6. The failure strain decreased with age, and there were statistical differences between groups 1, 2 and 3, 4, 5, 6, and between 3 and 6 (Figure 5D). Figures 6G,H show the scatter plots of the failure stress and the failure strain with age. The linear fitting equation is *σ*_*u*_ = 2.025–0.021 × age, *R* ^2^ = 0.474, and *ε*_*u*_ = 0.578–0.005 × age, *R* ^2^ = 0.517.

### 4.4 Comparison of fitting parameters in the different stretching directions

**FIGURE 7:**
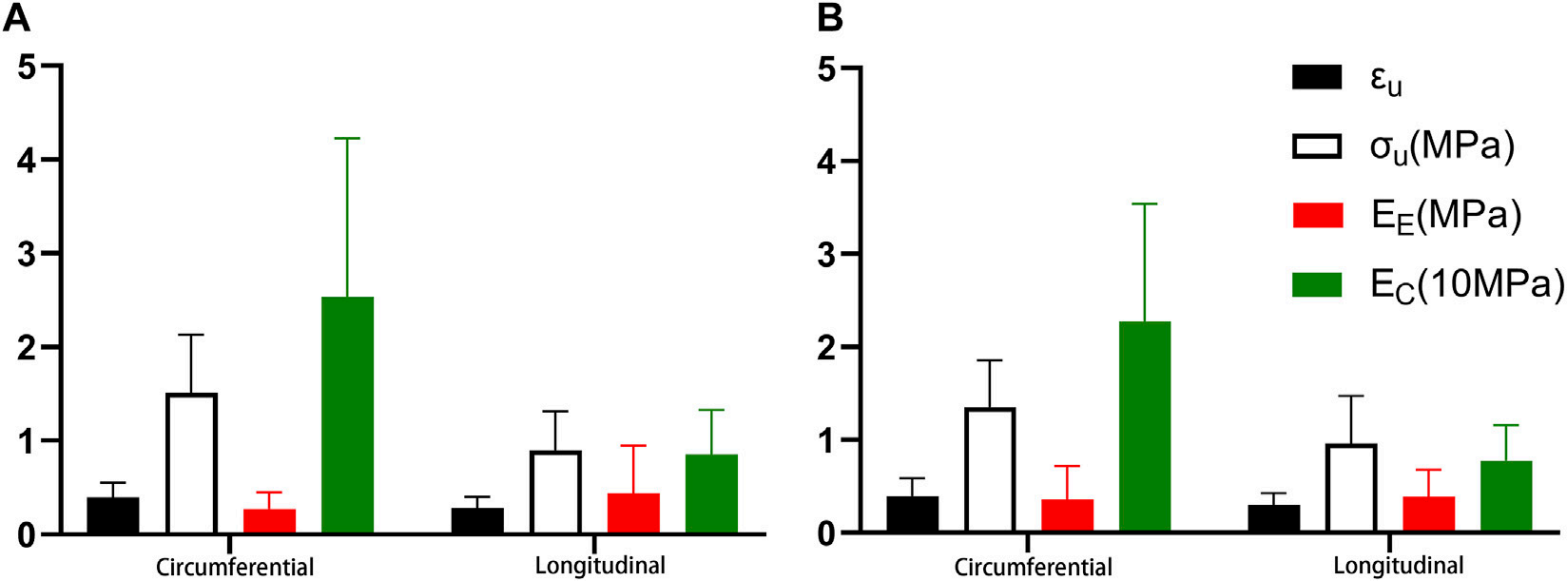
Comparison of the different stretching directions. **(A)**. Proximal segment; **(B)**. Distal segment.

There were statistical differences between proximal axial stretching and circumferential tensile, except *B*. *E*_*C*_, failure stress, and failure strain were greater under circumferential stretching than under axial stretching, while *E*_*E*_ was smaller. The mean circumferential tensile failure stress and failure strain were 1.511 MPa and 0.394, respectively; the mean *E*_*E*_ and *E*_*C*_ were 0.271 MPa and 25.335 MPa, respectively. The mean axial tensile failure stress and failure strain were 0.899 MPa and 0.279 MPa, respectively. The mean *E*_*E*_ and *E*_*C*_ were 0.440 MPa and 8.540 MPa, respectively (Figure 7A).

While there was no statistical difference in *E*_*E*_ between distal circumferential and axial stretching, there were statistical differences in other parameters. *E*_*c*_, failure stress, and failure strain were greater under circumferential stretching than under axial stretching. The mean circumferential tensile failure stress and failure strains were 1.350 MPa and 0.393 MPa respectively, and the mean *E*_*E*_ and *E*_*C*_ were 0.356 MPa and 22.776 MPa respectively. The mean axial tensile failure stress and failure strain were 0.960 MPa and 0.297, respectively, and the mean values of *E*_*E*_ and *E*_*C*_ were 0.386 MPa and 7.777 MPa, respectively (Figure 7B).

### 4.5 Comparison of fitting parameters in different segments

**FIGURE 8:**
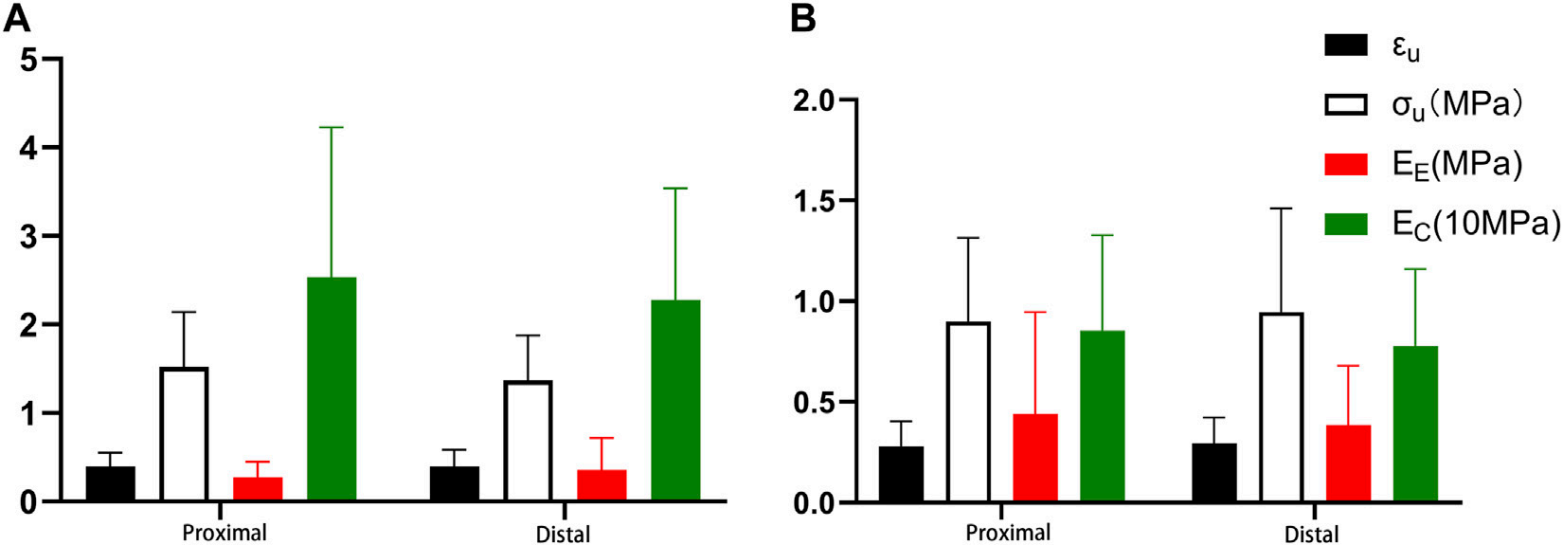
Comparison of different segments **(A)**. Circumferential tension; **(B)**. Axial tension.

There were no statistically significant differences in the fitting parameters and physiological moduli between the proximal and distal segments for both circumferential and axial stretching (Figure 8).

### 4.6 Comparing the effects of gender on mechanical parameters

**FIGURE 9:**
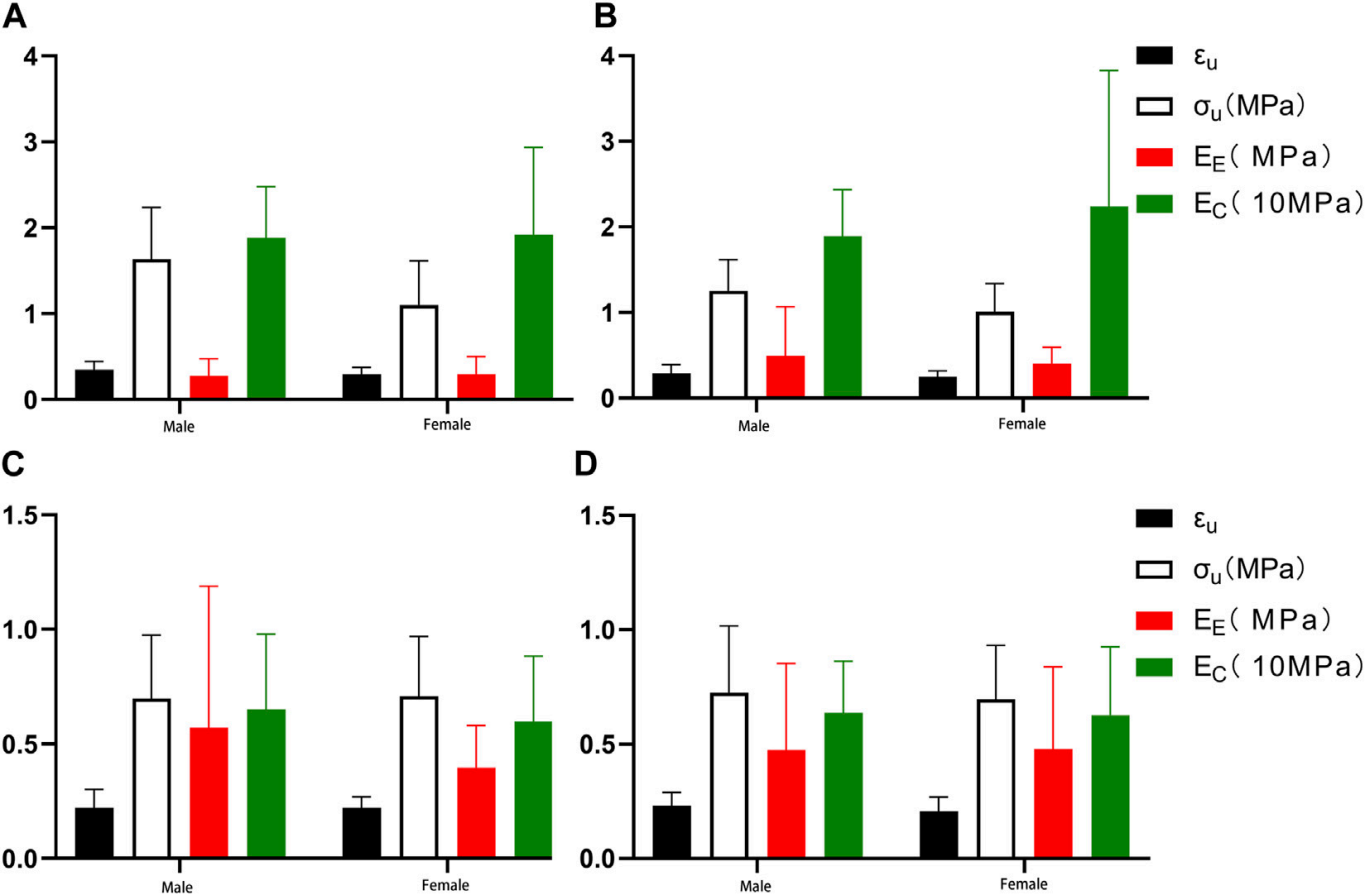
Comparing the effects of gender on mechanical parameters. **(A)** Proximal circumferential tensile; **(B)**. Distal circumferential tensile; **(C)**. Proximal axial tensile; **(D)**. Distal axial tensile.

To investigate the effect of gender on the mechanical properties of the aorta, we enrolled 23 patients (12 male and 11 female, mean age = 60.50 years for men and 59.36 years for women), namely, group 3, group 4, and group 5, aged 46–75 years. The fitting parameters and physiological moduli of the samples are listed in Tables 4 and 5. There was no statistical difference in the fitting parameters among the different gender groups. In proximal circumferential stretching, distal circumferential stretching, and distal axial stretching, the failure stress and the failure strain of the male group were all greater than those of the female group (Figure 9).

**TABLE 4:** Mean fitting parameters of different gender groups.

				Circumferential tension			Axial tension		
Group	N	Mean age	Segment	*K* (mm^2^/N)	*A*	*B* (MPa)	*K* (mm^2^/N)	*A*	*B* (MPa)
Male	12	60.50 ± 7.72 years	Proximal segment	0.058 ± 0.020	0.269 ± 0.103	0.065 ± 0.029	0.193 ± 0.140	0.117 ± 0.060	0.073 ± 0.072
			Distal segment	0.055 ± 0.016	0.228 ± 0.092	0.074 ± 0.037	0.166 ± 0.068	0.133 ± 0.046	0.059 ± 0.031
Female	11	59.36 ± 9.89 years	Proximal segment	0.069 ± 0.049	0.243 ± 0.094	0.063 ± 0.025	0.196 ± 0.102	0.104 ± 0.058	0.040 ± 0.022
			Distal segment	0.060 ± 0.034	0.201 ± 0.058	0.076 ± 0.029	0.191 ± 0.122	0.105 ± 0.053	0.046 ± 0.032

Note: Values are presented as the mean ± standard deviation.

**TABLE 5:** Mean physiological modulus of the different gender groups.

		Circumferential tension				Axial tension			
Group	Segment	*E*_*E*_ (MPa)	*E*_*C*_ (MPa)	*ε*_*u*_	*σ*_*u*_ (MPa)	*E*_*E*_ (MPa)	*E*_*C*_ (MPa)	*ε*_*u*_	*σ*_*u*_ (MPa)
Male	Proximal segment	0.279 ± 0.195	18.821 ± 5.976	0.348 ± 0.097	1.635 ± 0.599	0.571 ± 0.617	6.507 ± 3.283	0.222 ± 0.080	0.698 ± 0.277
	Distal segment	0.494 ± 0.575	18.944 ± 5.399	0.288 ± 0.103	1.254 ± 0.364	0.475 ± 0.378	6.372 ± 2.260	0.231 ± 0.058	0.726 ± 0.291
Female	Proximal segment	0.296 ± 0.207	19.232 ± 10.135	0.295 ± 0.081	1.101 ± 0.513	0.397 ± 0.183	5.981 ± 2.853	0.222 ± 0.047	0.708 ± 0.261
	Distal segment	0.402 ± 0.194	22.394 ± 15.873	0.249 ± 0.069	1.007 ± 0.331	0.479 ± 0.358	6.273 ± 2.982	0.207 ± 0.062	0.697 ± 0.235

Note: Values are presented as mean ± standard deviation.

### 4.7 Constitutive equations for the different segments in different age groups

We used the Levenberg–Marquardt minimization algorithm to fit the mean stress-strain curve for each group in the original data. The Fung-type strain energy function was used to fit the different segments from each age group. Root mean square difference *ε*) and correlation coefficient (*R* ^2^) were used to evaluate the fitting degree of the experimental data and the model (Table 6). The fitting degree of the constitutive equation for each segment was satisfactory.

**TABLE 6:** Constitutive equation parameters of the different segments in the different age groups.

Group	Segment	*K* (*mm* ^*2*^ */N*)	*C*_*θθ*_	*C*_*ZZ*_	*C*_*θZ*_	*ε*	*R* ^2^
27–35 years	Proximal segment	0.082	2.753	6.194	0.399	0.075	0.975
	Distal segment	0.082	5.896	12.084	13.492	0.044	0.990
36–45 years	Proximal segment	0.024	7.390	19.626	13.972	0.031	0.993
	Distal segment	0.069	4.171	7.732	8.204	0.070	0.973
46–55 years	Proximal segment	0.032	6.766	14.811	0.057	0.047	0.987
	Distal segment	0.051	8.201	12.283	0.142	0.036	0.987
56–65 years	Proximal segment	0.060	6.211	10.154	1.680	0.029	0.991
	Distal segment	0.020	13.921	18.735	2.804	0.030	0.982
66–75 years	Proximal segment	0.049	15.031	17.208	0.838	0.033	0.989
	Distal segment	0.037	28.941	13.056	2.419	0.016	0.996
76–86 years	Proximal segment	0.027	23.922	26.395	0.636	0.020	0.989
	Distal segment	0.039	26.787	17.259	0.990	0.028	0.993
All	Proximal segment	0.151	17.103	23.950	36.465	0.039	0.992
	Distal segment	0.067	7.786	6.911	0.230	0.035	0.988

## 5 Discussion

In this study, a total of 50 descending thoracic aortae from Chinese adults in forensic cases were subjected to a uniaxial tensile test. The study found that both model parameters and physiological moduli changed with age, which was consistent with previous studies (Haskett et al., 2010; Sokolis et al., 2017). This is the first systematic study that applies the uniaxial tensile test on the different segments of the Chinese adult descending thoracic aorta of different ages. It also has the largest size of uniaxial tensile samples of the human aorta (non-aortic aneurysm) so far (Mohan and Melvin, 1982; Perejda et al., 1985; Sherebrin et al., 1989; García-Herrera et al., 2012; Ninomiya et al., 2015). We have also worked out the constitutive equations for different ages and segments of the descending thoracic aorta (Table 6), which can be used for biomedical modeling.

### 5.1 The failure stress and the failure strain

In this study, the mean failure stress and failure strain under circumferential tensile of the proximal descending thoracic aorta were 1.511 MPa and 0.394, and those of axial tensile was 0.899 MPa and 0.279 respectively. The mean failure stress and failure strain of circumferential tensile of the distal descending thoracic aorta were 1.350 MPa and 0.393; their values were 0.960 MPa and 0.297 for axial tensile, respectively. Mohan and Melvin (Mohan and Melvin, 1982) reported the mean failure stress and failure strain under quasi-static uniaxial stretching in the middle part of the normal descending thoracic aorta for 19 cases (mean age 54.6 years), where mean values of circumferential stretching were 1.72 MPa and 0.53, and those of axial stretching were 1.47 MPa and 0.47. Both of these sets of values are slightly larger than those measured in this study. Ninomiya et al. (Ninomiya et al., 2015) performed a circumferential uniaxial tensile test on 26 aortas aged 33–89 years old (mean age = 64 years); the mean failure stress and failure strain of descending thoracic aorta were 1.688 MPa and 0.66, respectively. The failure strain was greater than that measured in this study. Sherebrin et al. (Sherebrin et al., 1989) performed a uniaxial tensile test on 23 descending thoracic aortas aged 15–81 years old and reported lower values with mean circumferential and axial failure stresses of 177 kPa and 184 kPa, respectively. Shah et al. (Shah et al., 2006) Performed dynamic biaxial tensile tests on 12 aortas and reported higher values with mean failure stress of 2.07 MPa and failure strain of 0.26 at 1 m/s. These differences may be related to the different ages and sites of samples or different experimental approaches.

This study also found that failure stress and failure strain of both circumferential and axial aortic stretching decreased with age (Figures 5, 6), which is consistent with previous study (Sherebrin et al., 1989). Changes in biomechanical characteristics of tissues are rarely considered in forensic age assessment. As shown in Figure 6, we conducted a linear regression fitting for the failure stress and failure strain in different tensile directions of different segments with age. Age-related changes in the failure stress and failure strain of the descending thoracic aorta might be used as auxiliary methods to evaluate the age of unknown cadavers in forensic practices.

### 5.2 Elastic modulus of elastic fiber and collagen fiber

Collagen fibers, elastic fibers, and smooth muscle cells are the main components of the aortic wall, which are directly related to its material properties (Sherebrin et al., 1989). Elastin is an elastic protein with mechanical behavior similar to Hookean material. Collagen fibers, which are stronger than elastic fibers, are arranged in a spiral pattern within the medial membrane of the artery wall. Elastic fibers are responsible for the elastic properties of the aorta, while collagen fibers provide mechanical strength (Sherebrin et al., 1989).

The contents and moduli of elastic and collagen fibers in the aortic wall vary widely in the literature. Ninomiya et al. (Ninomiya et al., 2015) found that the content of collagen fibers in the aortic wall was positively correlated with and increased with age, while the elastic fibers did not. Wuyts et al. (Wuyts et al., 1995) proposed a circumferential stress-strain mathematical model based on the vascular wall structure and fitted the aortic stretching data in the literature by Langewouters et al. (Langewouters et al., 1984). The mean value of the elastic modulus of thoracic aortic elastic fibers calculated by model parameters was 0.532 MPa, and the mean value of the elastic modulus of collagen fiber was 300 MPa. The elastic modulus of elastic fiber decreases with age, while that of collagen fiber increases. By establishing the strain energy function model of the aorta, Zulliger et al. (Zulliger and Stergiopulos, 2007) analyzed the data in the literature of Langewouters et al. (Langewouters et al., 1984). They fixed the elastic modulus of collagen fiber at 200 MPa and found the mean elastic modulus of elastic fiber was 0.06 MPa. It was concluded that the elastic modulus of the aortic elastic fibers is not age-related, and the main reason for the decrease in aortic elasticity is a change in the collagen fiber network with age, which affects the aortic mechanical properties.

This study calculated the elastic modulus of elastic fibers and collagen fibers to be 0.271 MPa and 25.335 MPa in the circumferential direction of the proximal end, and 0.440 MPa and 8.540 MPa in the axial direction. The circumferential and axial stretches of the distal end were 0.356 MPa and 22.776 MPa, and 0.386 MPa and 7.777 MPa, respectively. As shown in Figure 4, the elastic modulus of elastic fibers increases with age, while the elastic modulus of collagen fibers decreases with age. This may be due to the loss of elastic fibers with age, and the earlier involvement of collagen fibers in mechanical bearing, resulting in an increase in the modulus of elastic fibers calculated by the model with age. For collagen fibers, this study calculated the maximum modulus from stretching to fracture. As the age increases, the failure stress and strain also decrease. The final result is that the slope of the stress-strain curve before failure increases with age. Therefore, the calculated collagen fiber modulus is smaller than that in the literature, and it decreases with age.

### 5.3 Comparison of mechanical parameters of circumferential and axial tensions

In this study, the mean failure strain, failure stress, and elastic modulus of collagen fiber of each group in circumferential stretching were larger than that of axial stretching. In addition, as shown in Figure 7, the elastic modulus of the elastic fiber was smaller than that of axial stretching (no significant difference at the distal segment). This is consistent with most studies in the literature (Langewouters et al., 1984).

The aorta is composed of intima, media, and adventitia. The mechanical response of aortic anisotropy is due to the different alignment of elastic fibers and collagen fibers (Sokolis, 2023). Both fiber families are approximately circumferential in the media, longitudinal in the adventitia, and relatively evenly distributed in the intima (Sokolis, 2023). Therefore, its response is orthotropic and can be considered to originate from fiber-reinforced material (Holzapfel et al., 2000; Holzapfel, 2006). The circumferential stretching of the arterial wall has larger values of failure strain and failure stress; the elastic modulus of collagen fibers is also higher. The elastic modulus of elastic fiber in circumferential stretching is smaller than that in axial stretching, which may be related to the involvement of passive loading of collagen fiber in axial stretching. Thus, a circumferential bias of fibers orientation in the media is the basis for the anisotropic behavior of the aorta. Haskett et al. (Haskett et al., 2010)found that the fiber direction of the aorta is mainly circumferential at all positions and ages. With increasing age, both circumferential and axial fiber stiffness will increase, making it easier for the aorta to fail.

### 5.4 Comparison of mechanical parameters of the proximal and distal aorta

Currently, no studies exist on the comparison of different parts of the human descending thoracic aorta using the uniaxial tensile test. In most relevant studies (Mohan and Melvin, 1982; Sherebrin et al., 1989; García-Herrera et al., 2012; Ninomiya et al., 2015), the mechanical properties of different parts of the descending thoracic aorta are presumed to be isotropic. Ninomiya et al. (Ninomiya et al., 2015) conducted a circumferential uniaxial tensile test on the thoracic and abdominal aorta and found that the descending thoracic aorta had higher strength and elasticity than the abdominal aorta. In this study, the descending thoracic aorta was divided into the proximal and the distal segments; circumferential and axial uniaxial tensile tests were performed, respectively, to calculate the model parameters, failure strain, failure stress, as well as the elastic modulus of elastic fiber and collagen fiber. No statistical differences were observed between the proximal and distal descending thoracic aorta (Figure 8).

### 5.5 Comparison of mechanical parameters of the descending thoracic aorta in different genders

Gender differences in the occurrence, development, treatment, and prognosis of cardiovascular diseases have attracted more and more attention (Chung et al., 2020). Although women are less likely to develop thoracic aortic aneurysms, they are three times more likely than men to have a dissection or rupture (Davies et al., 2002). The risk of rupture and death in women is 4 times higher than that in men (Dimick et al., 2002; Brown et al., 2003), and the incidence of postoperative complications is nearly 3 times greater than that in men (Wolf et al., 2002). It can be seen that although aortic aneurysm is more common in men, female aortic aneurysm patients have a higher risk of rupture and a worse clinical prognosis. The underlying mechanism of this gender difference is still not fully understood.

Our study showed that the failure stress and failure strain in the male group were greater than in the female group. This was especially true for proximal circumferential stretching, distal circumferential stretching, and distal axial stretching. There were statistically significant differences in the failure stresses of proximal circumferential tensile, while no statistically significant differences were noted for others (Figure 9). This is similar to the results of Ninomiya et al. (Ninomiya et al., 2015), who conducted circumferential uniaxial tensile tests on the descending thoracic aorta and abdominal aorta of 9 males and 9 females. Although dissection is more frequent in males than in females, Sokolis et al. (Sokolis and Papadodima, 2022) found that there is no gender-related effect on the delamination properties of the aorta. In terms of circumferential residual strain, females and males have similar opening angle and residual stretches (Sokolis et al., 2017). Sokolis et al. (Sokolis and Iliopoulos, 2014) provides a molecular explanation for this gender difference. Higher levels of matrix metalloproteinase (MMP)-2 and MMP-9, which can degrade and reconstruct arterial walls, and lower expression of tissue inhibitors of metalloproteinase (TIMP-1) and TIMP-2 in the females than in the male, may lead to increased degradation and decreased strength of the aortic extracellular matrix in females. Gender differences in aortic biomechanics may contribute to the greater risk of aortic aneurysm rupture and worse clinical prognosis in women than in men.

### 5.6 Limitations

The aortic wall is anisotropic and should ideally stretch in multiple directions simultaneously to mimic physiological conditions rather than with uniaxial tension. However, biaxial tension, for example, puts a higher demand on the specimen and equipment, and it is difficult to detect the failure of the specimen due to the small range of strain measurement. Therefore, uniaxial tension is still the dominant method for performing aortic mechanical tests.

Collecting human aorta is challenging, and obtaining an ideal age and gender distribution is difficult. The six age groups were not matched by gender. Futhermore, there were no samples for infants and adolescents under 26 years old in our study. The material properties of the aorta in infants and adolescents may differ from those of adults. We will thus continue to collect aortic specimens of different age group and perform further tensile tests.

Our study focused on the mechanical behavior of healthy aortas and excluded the arterial plaque and the aortic ostia. In the future, we will compare and study the biomechanical differences between the arterial tissue at the atherosclerotic site, tissue at the ostia, and the healthy aortic tissue, to provide data for abnormal aortic mechanical parameters.

## 6 Conclusion

Our study is the first uniaxial tensile test on the adult descending thoracic aorta of different ages and segments. It has the largest sample size of the human aorta (non-aortic aneurysm) subjected to uniaxial tensile tests so far. The model parameters, failure stress, failure strain, elastic modulus of elastic fiber, and elastic modulus of collagen fiber in different thoracic aortic segments of 50 adults were obtained, and the differences between groups and gender were statistically analyzed. Additionally, the Fung hyperelastic constitutive equations of the different segments of the descending thoracic aorta in different age groups were fitted, which can be used by biomedical engineers for modeling.

## Data Availability

The original contributions presented in the study are included in the article/Supplementary Material, further inquiries can be directed to the corresponding authors.

## References

[B1] Bass C. R. Darvish K. Bush B. Crandall J. R. Srinivasan S. C. Tribble C. (2001). Material properties for modeling traumatic aortic rupture. Stapp Car Crash J. 45, 143–160. 10.4271/2001-22-0006 17458743

[B2] Brown P. M. Zelt D. T. Sobolev B. (2003). The risk of rupture in untreated aneurysms: The impact of size, gender, and expansion rate. J. Vasc. Surg. 37 (2), 280–284. 10.1067/mva.2003.119 12563196

[B3] Chung J. Coutinho T. Chu M. W. Ouzounian M. (2020). Sex differences in thoracic aortic disease: A review of the literature and a call to action. J. Thorac. Cardiovasc. Surg. 160 (3), 656–660. 10.1016/j.jtcvs.2019.09.194 31987620

[B4] Chuong C. J. Fung Y. C. (1983). Three-dimensional stress distribution in arteries. J. biomechanical Eng. 105 (3), 268–274. 10.1115/1.3138417 6632830

[B5] Davies R. R. Goldstein L. J. Coady M. A. Tittle S. L. Rizzo J. A. Kopf G. S. (2002). Yearly rupture or dissection rates for thoracic aortic aneurysms: Simple prediction based on size. Ann. Thorac. Surg. 73 (1), 17–28. 10.1016/s0003-4975(01)03236-2 11834007

[B6] Dimick J. B. Stanley J. C. Axelrod D. A. Kazmers A. Henke P. K. Jacobs L. A. (2002). Variation in death rate after abdominal aortic aneurysmectomy in the United States: Impact of hospital volume, gender, and age. Ann. Surg. 235 (4), 579–585. 10.1097/00000658-200204000-00017 11923615 PMC1422474

[B7] Ferrara A. Morganti S. Totaro P. Mazzola A. Auricchio F. (2016). Human dilated ascending aorta: Mechanical characterization via uniaxial tensile tests. J. Mech. Behav. Biomed. Mater. 53, 257–271. 10.1016/j.jmbbm.2015.08.021 26356765

[B8] Ferrara A. Totaro P. Morganti S. Auricchio F. (2018). Effects of clinico-pathological risk factors on *in-vitro* mechanical properties of human dilated ascending aorta. J. Mech. Behav. Biomed. Mater. 77, 1–11. 10.1016/j.jmbbm.2017.08.032 28886508

[B9] García-Herrera C. M. Atienza J. M. Rojo F. J. Claes E. Guinea G. V. Celentano D. J. (2012). Mechanical behaviour and rupture of normal and pathological human ascending aortic wall. Med. Biol. Eng. Comput. 50 (6), 559–566. 10.1007/s11517-012-0876-x 22391945

[B10] Grootenboer N. Bosch J. Hendriks J. van Sambeek M. (2009). Epidemiology, aetiology, risk of rupture and treatment of abdominal aortic aneurysms: Does sex matter? Eur. J. Vasc. Endovascular Surg. 38 (3), 278–284. 10.1016/j.ejvs.2009.05.004 19540779

[B11] Guinea G. V. Atienza J. M. Rojo F. J. García-Herrera C. M. Yiqun L. Claes E. (2010). Factors influencing the mechanical behaviour of healthy human descending thoracic aorta. Physiol. Meas. 31 (12), 1553–1565. 10.1088/0967-3334/31/12/001 20980717

[B12] Hans S. S. Jareunpoon O. Balasubramaniam M. Zelenock G. B. (2005). Size and location of thrombus in intact and ruptured abdominal aortic aneurysms. J. Vasc. Surg. 41 (4), 584–588. 10.1016/j.jvs.2005.01.004 15874920

[B13] Haskett D. Johnson G. Zhou A. Utzinger U. Vande Geest J. (2010). Microstructural and biomechanical alterations of the human aorta as a function of age and location. Biomechanics Model. Mechanobiol. 9 (6), 725–736. 10.1007/s10237-010-0209-7 20354753

[B14] Hiller R. J. Mikocka-Walus A. A. Cameron P. A. (2010). Aortic transection: Demographics, treatment and outcomes in victoria, Australia. Emerg. Med. J. 27 (5), 368–371. 10.1136/emj.2009.075978 20442166

[B15] Holzapfel G. A. (2006). Determination of material models for arterial walls from uniaxial extension tests and histological structure. J. Theor. Biol. 238 (2), 290–302. 10.1016/j.jtbi.2005.05.006 16043190

[B16] Holzapfel G. A. Gasser T. C. Ogden R. W. (2000). A new constitutive framework for arterial wall mechanics and a comparative study of material models. J. Elast. Phys. Sci. solids 61 (1), 1–48. 10.1023/a:1010835316564

[B17] Iliopoulos D. C. Kritharis E. P. Boussias S. Demis A. Iliopoulos C. D. Sokolis D. P. (2013). Biomechanical properties and histological structure of sinus of Valsalva aneurysms in relation to age and region. J. biomechanics 46 (5), 931–940. 10.1016/j.jbiomech.2012.12.004 23332823

[B18] Kent K. C. (2014). Abdominal aortic aneurysms. N. Engl. J. Med. 371 (22), 2101–2108. 10.1056/nejmcp1401430 25427112

[B19] Langewouters G. J. Wesseling K. H. Goedhard W. (1984). The static elastic properties of 45 human thoracic and 20 abdominal aortas *in vitro* and the parameters of a new model. J. biomechanics 17 (6), 425–435. 10.1016/0021-9290(84)90034-4 6480618

[B20] Lederle F. A. Wilson S. E. Johnson G. R. Reinke D. B. Littooy F. N. Acher C. W. (2002). Immediate repair compared with surveillance of small abdominal aortic aneurysms. N. Engl. J. Med. 346 (19), 1437–1444. 10.1056/nejmoa012573 12000813

[B21] Mohan D. Melvin J. W. (1982). Failure properties of passive human aortic tissue. I—uniaxial tension tests. J. biomechanics 15 (11), 887–902. 10.1016/0021-9290(82)90055-0 7161291

[B22] Neschis D. G. Scalea T. M. Flinn W. R. Griffith B. P. (2008). Blunt aortic injury. N. Engl. J. Med. 359 (16), 1708–1716. 10.1056/nejmra0706159 18923173

[B23] Ninomiya O. H. Tavares Monteiro J. A. Higuchi M. d. L. Puech-Leão P. de Luccia N. Raghavan M. L. (2015). Biomechanical properties and microstructural analysis of the human nonaneurysmal aorta as a function of age, gender and location: An autopsy study. J. Vasc. Res. 52 (4), 257–264. 10.1159/000442979 26799837

[B24] Pei M. Zou D. Gao Y. Zhang J. Huang P. Wang J. (2021). The influence of sample geometry and size on porcine aortic material properties from uniaxial tensile tests using custom-designed tissue cutters, clamps and molds. PLoS One 16 (2), e0244390. 10.1371/journal.pone.0244390 33556052 PMC7869995

[B25] Perejda A. J. Abraham P. A. Carnes W. H. Coulson W. F. Uitto J. (1985). Marfan's syndrome: Structural, biochemical, and mechanical studies of the aortic media. J. laboratory Clin. Med. 106 (4), 376–383. 4045295

[B26] Pichamuthu J. E. Phillippi J. A. Cleary D. A. Chew D. W. Hempel J. Vorp D. A. (2013). Differential tensile strength and collagen composition in ascending aortic aneurysms by aortic valve phenotype. Ann. Thorac. Surg. 96 (6), 2147–2154. 10.1016/j.athoracsur.2013.07.001 24021768 PMC4016718

[B27] Raghavan M. L. Hanaoka M. M. Kratzberg J. A. Higuchi M. d. L. da Silva E. S. (2011). Biomechanical failure properties and microstructural content of ruptured and unruptured abdominal aortic aneurysms. J. biomechanics 44 (13), 2501–2507. 10.1016/j.jbiomech.2011.06.004 21763659

[B28] Raghavan M. L. Webster M. W. Vorp D. A. (1996). *Ex vivo* biomechanical behavior of abdominal aortic aneurysm: Assessment using a new mathematical model. Ann. Biomed. Eng. 24 (5), 573–582. 10.1007/bf02684226 8886238

[B29] Sacks M. S. Sun W. (2003). Multiaxial mechanical behavior of biological materials. Annu. Rev. Biomed. Eng. 5 (1), 251–284. 10.1146/annurev.bioeng.5.011303.120714 12730082

[B30] Shah C. S. Hardy W. N. Mason M. J. Yang K. H. Van Ee C. A. Morgan R. (2006). Dynamic biaxial tissue properties of the human cadaver aorta. Stapp Car Crash J. 50, 217–246. 10.4271/2006-22-0010 17311166

[B31] Sherebrin M. H. Hegney J. E. Roach M. R. (1989). Effects of age on the anisotropy of the descending human thoracic aorta determined by uniaxial tensile testing and digestion by NaOH under load. Can. J. physiology Pharmacol. 67 (8), 871–878. 10.1139/y89-136 2598122

[B32] Sokolis D. P. Gouskou N. Papadodima S. A. Kourkoulis S. K. (2021). Layer-specific residual deformations and their variation along the human aorta. J. Biomechanical Eng. 143 (9), 094504. 10.1115/1.4050913 33876198

[B33] Sokolis D. P. Iliopoulos D. C. (2014). Impaired mechanics and matrix metalloproteinases/inhibitors expression in female ascending thoracic aortic aneurysms. J. Mech. Behav. Biomed. Mater. 34, 154–164. 10.1016/j.jmbbm.2014.02.015 24583920

[B34] Sokolis D. P. Kritharis E. P. Giagini A. T. Lampropoulos K. M. Papadodima S. A. Iliopoulos D. C. (2012). Biomechanical response of ascending thoracic aortic aneurysms: Association with structural remodelling. Comput. methods biomechanics Biomed. Eng. 15 (3), 231–248. 10.1080/10255842.2010.522186 21480082

[B35] Sokolis D. P. (2023). Layer-specific tensile strength of the human aorta: Segmental variations. J. Biomechanical Eng. 145 (6), 064502. 10.1115/1.4056748 36691824

[B36] Sokolis D. P. Papadodima S. A. (2022). Regional delamination strength in the human aorta underlies the anatomical localization of the dissection channel. J. Biomechanics 141, 111174. 10.1016/j.jbiomech.2022.111174 35701262

[B37] Sokolis D. P. (2007). Passive mechanical properties and structure of the aorta: Segmental analysis. Acta physiol. 190 (4), 277–289. 10.1111/j.1748-1716.2006.01661.x 17635348

[B38] Sokolis D. P. Savva G. D. Papadodima S. A. Kourkoulis S. K. (2017). Regional distribution of circumferential residual strains in the human aorta according to age and gender. J. Mech. Behav. Biomed. Mater. 67, 87–100. 10.1016/j.jmbbm.2016.12.003 27988442

[B39] Sulejmani F. Pokutta-Paskaleva A. Ziganshin B. Leshnower B. Iannucci G. Elefteriades J. (2017). Biomechanical properties of the thoracic aorta in Marfan patients. Ann. Cardiothorac. Surg. 6 (6), 610–624. 10.21037/acs.2017.09.12 29270373 PMC5721111

[B40] Teixeira P. G. Inaba K. Barmparas G. (2011). Blunt thoracic aortic injuries: An autopsy study. J. Trauma 70 (1), 581–202. 10.1016/j.jvs.2011.06.087 21217494

[B41] Vallabhaneni S. R. Gilling-Smith G. L. How T. V. Carter S. D. Brennan J. A. Harris P. L. (2004). Heterogeneity of tensile strength and matrix metalloproteinase activity in the wall of abdominal aortic aneurysms. J. Endovascular Ther. 11 (4), 494–502. 10.1583/04-1239.1 15298501

[B42] Vito R. P. Dixon S. A. (2003). Blood vessel constitutive models—1995–2002. Annu. Rev. Biomed. Eng. 5 (1), 413–439. 10.1146/annurev.bioeng.5.011303.120719 12730083

[B43] Vorp D. A. Schiro B. J. Ehrlich M. P. Juvonen T. S. Ergin M. Griffith B. P. (2003). Effect of aneurysm on the tensile strength and biomechanical behavior of the ascending thoracic aorta. Ann. Thorac. Surg. 75 (4), 1210–1214. 10.1016/s0003-4975(02)04711-2 12683565

[B44] Watanabe K. Fukuda I. Asari Y. (2013). Management of traumatic aortic rupture. Surg. today 43, 1339–1346. 10.1007/s00595-012-0471-7 23338596

[B45] Wolf Y. G. Arko F. R. Hill B. B. Olcott C. Harris E. Fogarty T. J. (2002). Gender differences in endovascular abdominal aortic aneurysm repair with the AneuRx stent graft. J. Vasc. Surg. 35 (5), 882–886. 10.1067/mva.2002.123754 12021702

[B46] Wuyts F. L. Vanhuyse V. J. Langewouters G. J. Decraemer W. F. Raman E. R. Buyle S. (1995). Elastic properties of human aortas in relation to age and atherosclerosis: A structural model. Phys. Med. Biol. 40 (10), 1577–1597. 10.1088/0031-9155/40/10/002 8532741

[B47] Zulliger M. A. Stergiopulos N. (2007). Structural strain energy function applied to the ageing of the human aorta. J. biomechanics 40 (14), 3061–3069. 10.1016/j.jbiomech.2007.03.011 17822709

