# Supplementary figures and images for: A study to characterize the mechanical properties and material constitution of adult descending thoracic aorta based on uniaxial tensile test and digital image correlation
Source: Front Bioeng Biotechnol. 2023 Jun 14;11:1178199. doi: 10.3389/fbioe.2023.1178199 (PMC10306407; doi:10.3389/fbioe.2023.1178199)

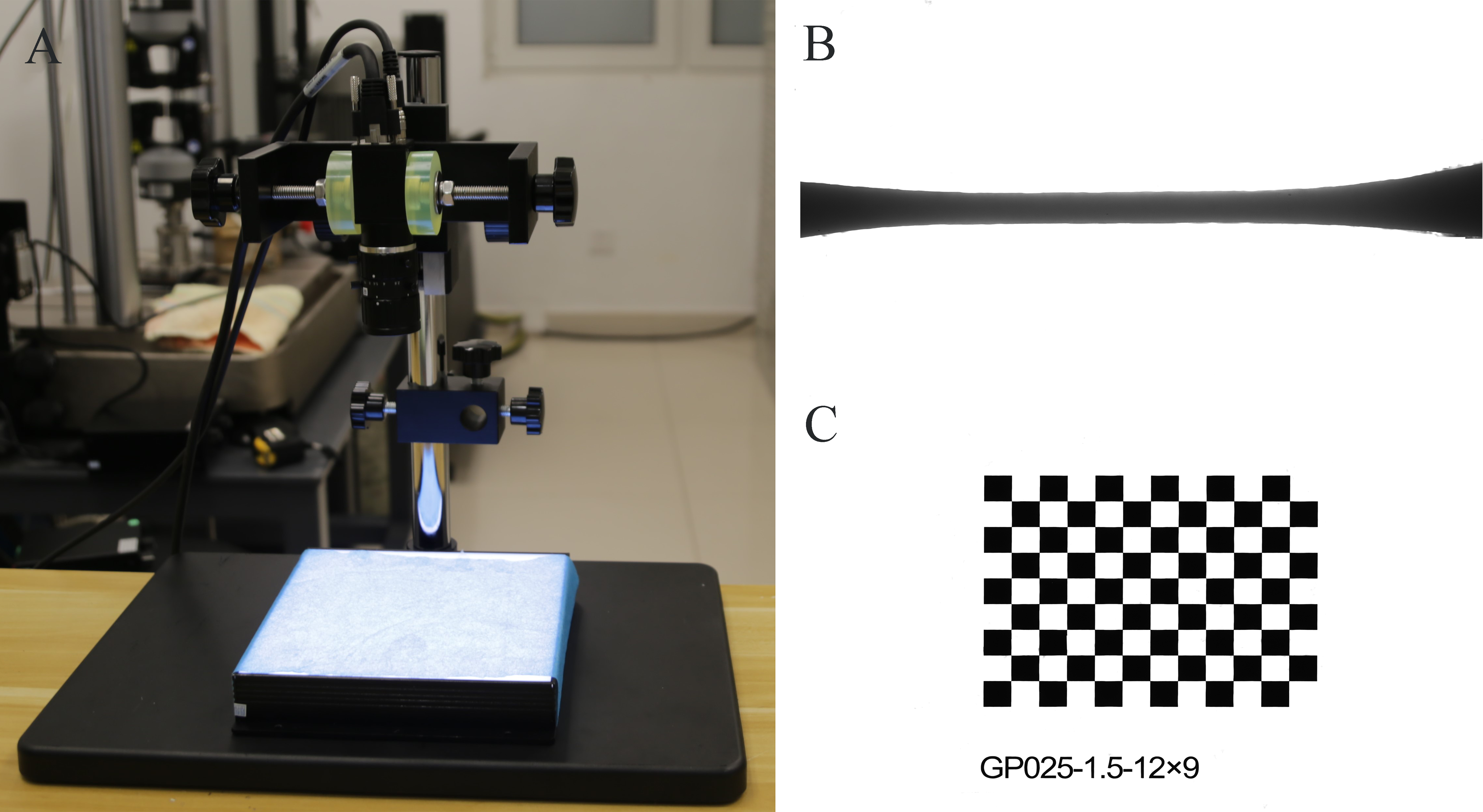

Supplement: Supplementary file 1 [file Image3.TIF]

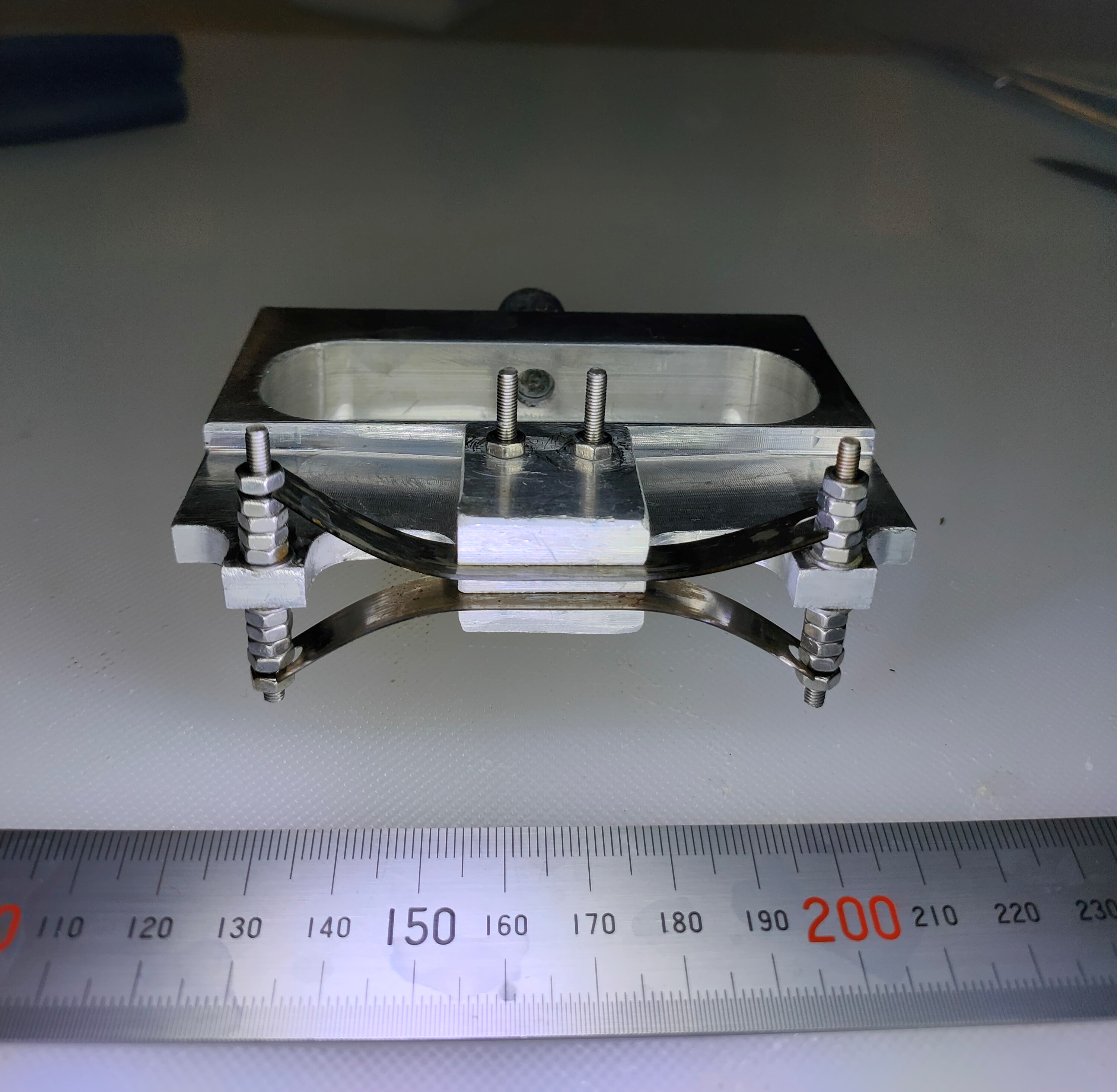

Supplement: Supplementary file 2 [file Image1.JPEG]

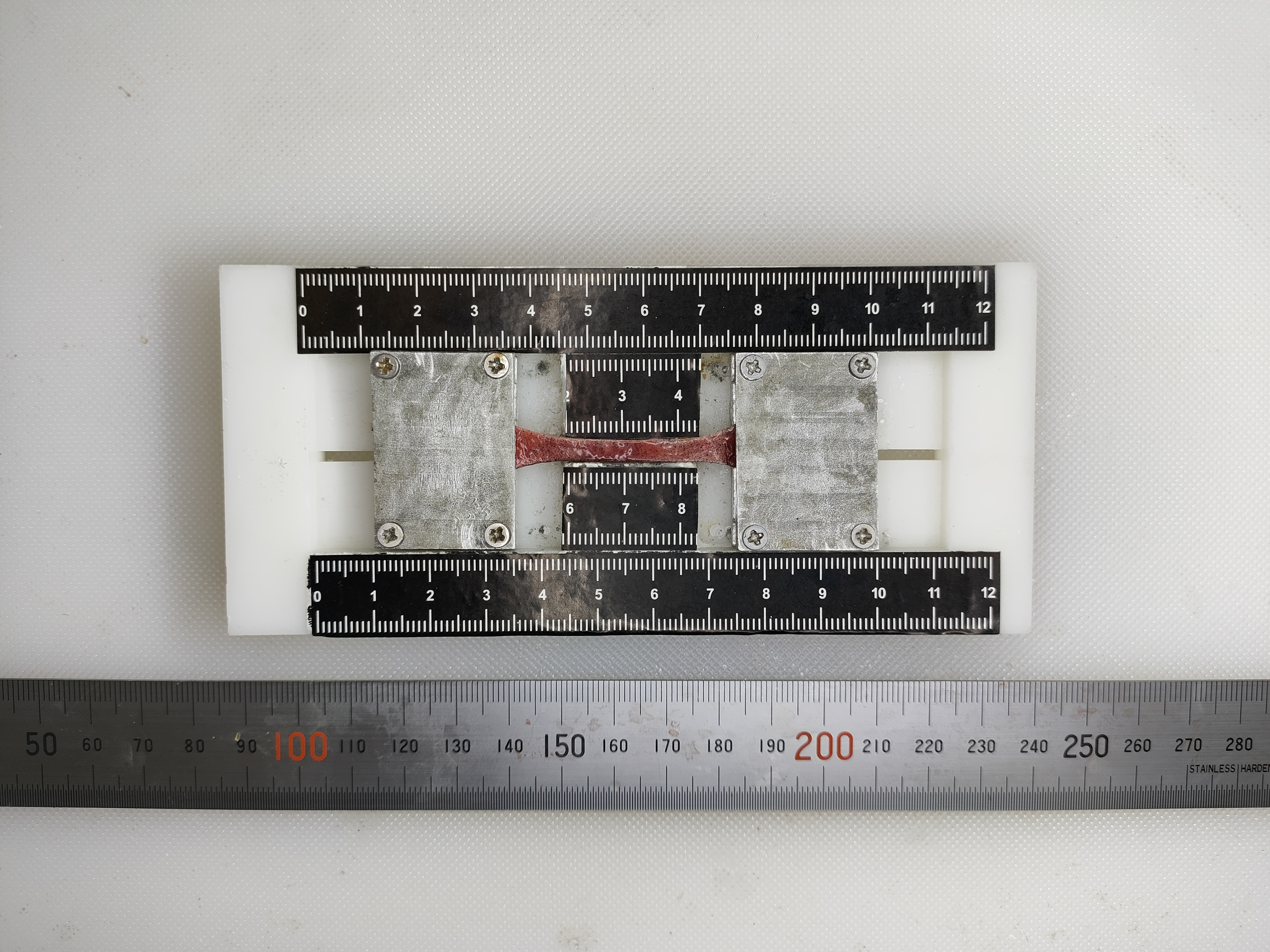

Supplement: Supplementary file 3 [file Image4.JPEG]

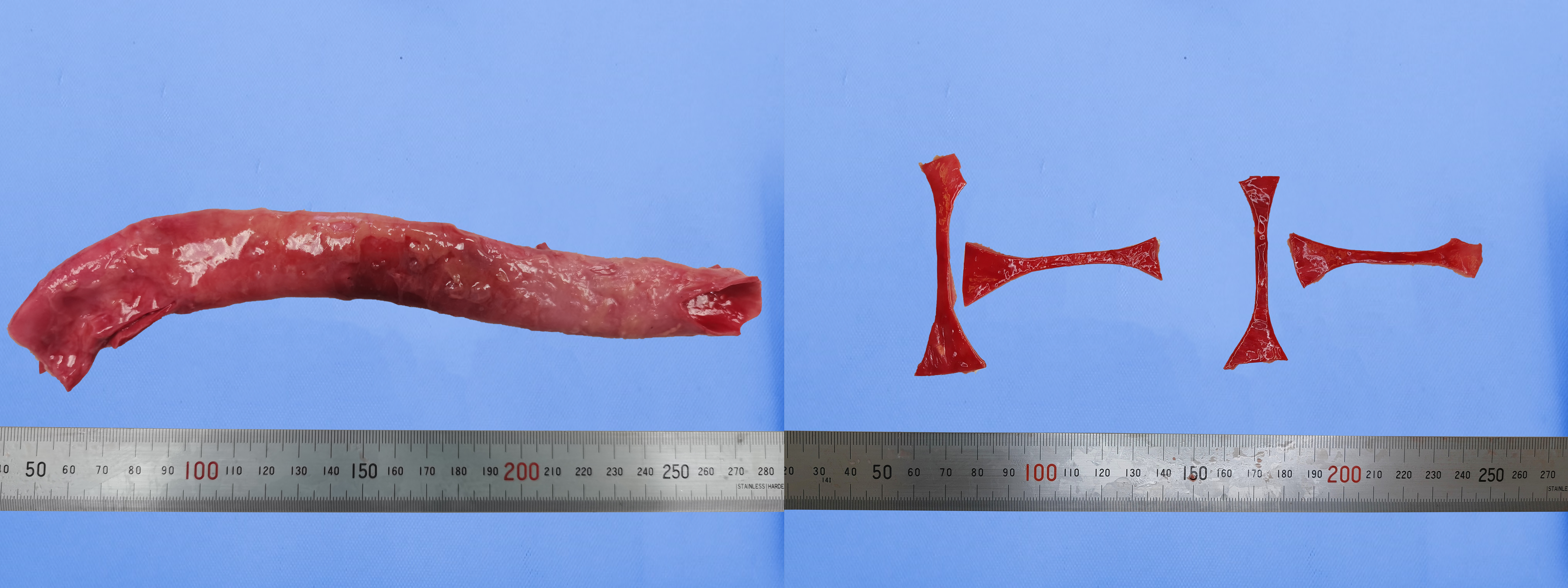

Supplement: Supplementary file 4 [file Image2.TIF]
